# Cell membrane-encased thylakoid as white light triggered PDT therapy for facile and targeted choroidal melanoma treatment

**DOI:** 10.1016/j.bioactmat.2026.03.058

**Published:** 2026-04-01

**Authors:** Jiahao Wang, Zhirong Chen, Yuexin Yang, Yajia Wang, Hongying Xie, Wenxin Hong, Quankui Lin

**Affiliations:** National Engineering Research Center of Ophthalmology and Optometry, School of Biomedical Engineering, School of Ophthalmology and Optometry, Eye Hospital, Wenzhou Medical University, Wenzhou, 325027, China

**Keywords:** Thylakoid, Photodynamic therapy, Cell membrane targeting, Choroidal melanoma, ROS

## Abstract

Choroidal melanoma, the most prevalent subtype of uveal melanoma with a bleak prognosis, necessitates safer and more efficient treatment methods. Tumor cell membrane-modified nanodelivery systems have exhibited significant enhancement in photodynamic therapy (PDT). Currently, photosensitizers utilized in PDT encompass porphyrin-related organic molecules and other types, requiring complex and intricate systems construction. This study focuses on harnessing the naturally occurring photodynamic active photosynthetic unit-thylakoid which serve as a major source of singlet oxygen. Initially, thylakoids extracted from fresh leaves were identified based on their absorbance value, morphology, and characteristic proteins. The tumor cell membrane-thylakoid membrane (CM-Thy) photodynamic therapy system was prepared through ultrasonic fragmentation and liposomal extrusion technology. Due to its membrane targeting ability, CM-Thy demonstrated excellent uptake performance towards OCM-1 cells within intraocular tissues. Both in vitro and in vivo experiments confirmed CM-Thy's photodynamic activity. Specifically, Thy in the system generates singlet oxygen, after cellular internalization, the elevated intracellular reactive oxygen species (ROS) triggered by this process induce cell lipid peroxidation and increased membrane permeability, while singlet oxygen further mediates DNA oxidative damage, reduced cell proliferation capacity, and NLRP3 inflammasome-mediated pyroptosis, all these effects synergistically contribute to ultimate tumor cell apoptosis. Furthermore, the anti-tumor effect of CM-Thy was validated through various mechanisms involved in tumor formation such as angiogenesis and vasculogenic mimicry. Interestingly, the results from visible light experiments conducted in vitro also substantiated the remarkable therapeutic efficacy of this system for refractive eye disorders while providing innovative ideas for cross-species biological interventions.

## Highlights

• A biomimetic photodynamic therapy (PDT) system (CM-Thy) was constructed by fusing tumor cell membranes (CM) with plant-derived thylakoids (Thy), enabling homologous targeting and efficient PDT for choroidal melanoma.
• Thy in CM-Thy generates abundant singlet oxygen (^1^O_2_) upon white light or 660 nm laser irradiation, inducing tumor cell apoptosis via dual mechanisms: direct DNA oxidative damage and NLRP3 inflammasome-mediated pyroptosis.
• CM-Thy inhibits choroidal melanoma progression by targeting multiple tumorigenic mechanisms, including angiogenesis, vasculogenic mimicry, cell migration, and 3D tumor spheroid formation.
• Activation of CM-Thy by natural daily light provides a mild, non-invasive therapeutic strategy, opening new avenues for cross-species biological interventions in ocular tumors.

## Graphical abstract

The nanocomposites of cell membrane-thylakoid (CM-Thy) are prepared through extrusion from the mixture of spinach's thylakoids and tumor cells' membranes. Injection of the membrane complexes into the subretinal space has demonstrated remarkable therapeutic effects in treating choroidal melanoma. In this process of tumor eradication, the cell membrane facilitates targeted uptake of the complex, while the photodynamic therapeutic property of thylakoid is activated by visible light, including daily light sources, leading to inhibitory effects on various mechanisms involved in tumor formation.

**Figure:**
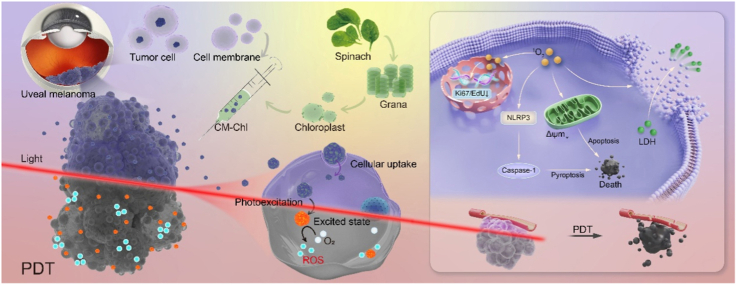

## 1 Introduction

Malignant tumors pose a significant threat to human life and health worldwide. The uncontrolled proliferation of internal cells and abnormal vascular structure promote the invasion and metastasis of malignant tumors [1,2]. Uveal melanoma is the most prevalent primary intraocular tumor in adults, with approximately 90% occurring in the choroid [3,4]. Clinical treatment for this disease primarily focuses on local tumor control and reducing the risk of metastasis through methods such as enucleation, local tumor resection, radiation therapy, laser therapy, and other therapies [5,6]. Despite achieving local tumor control through surgical resection combined with radiotherapy, distant metastases can still occur in 50% of patients [7].

In recent years, scholars have discussed combination therapies based on biological therapy for treating malignant tumors. Various techniques such as chemotherapy, photothermal therapy, photodynamic therapy, and immunotherapy have been explored [8]. Photodynamic therapy has been successfully and widely applied in extensive experimental animal studies as a main and highly promising treatment method for the challenging and complex condition of choroidal melanoma [9,10].

This concept breaks through traditional anticancer limitations by overcoming oncological barriers and effectively utilizing drug properties. The cell membrane-modified bionic nanodrug delivery system combines natural cell membranes with functional nanosystems to provide advantages like low immunogenicity, extended circulation time, specific targeting ability, and high biocompatibility [11].

However, tumor cells' infinite proliferative capacity makes them an important source for obtaining large quantities of cell membranes required for bionic nano-drug delivery systems via in vitro culture [12]. The surface of tumor cell membranes contains various functional molecules closely associated with homologous binding abilities such as N-cadherin galectin-3 and epithelial cell adhesion molecules (EpCAM) [13]. As a crucial cellular structure involved in mutual recognition and regulation between different cells within the body's ecosystem, the cell membrane plays a vital role in protection [14].

As far as eye tumors are concerned, the eyeball itself serves as an excellent refractive system, which undoubtedly provides a natural advantage for PDT, is a non-invasive, highly selective, controllable treatment that efficiently removes excess cells and tissues while easily combining with other treatments [15,16]. PDT has received approval from the United States Food and Drug Administration (FDA) and has subsequently been applied in clinical treatment [17]. It is considered one of the most effective therapies for both tumor and non-malignant diseases. The commonly used methods for PDT include intravenous injection or local application of photosensitizer to achieve therapeutic effects [18]. Photodynamic efficacy refers to the use of specific wavelengths to stimulate light and activate the photosensitizer when it accumulates in target cells and tissues [19,20]. The photosensitizer undergoes a series of energy transitions, initially forming an excited singlet state followed by a long-lived excited triplet state, leading to photochemical reactions that generate singlet oxygen (^1^O_2_) and other ROS on-site [[21], [22], [23]]. ROS selectively eliminates unsuitable cells, pathogenic microorganisms, and tissues to destroy lesions and treat diseases effectively [24]. PDT has been proven to have significant inhibitory effects on malignant ophthalmic tumors such as choroidal melanoma with low recurrence rates [25,26].

In nature, it has long been recognized that chloroplasts in plants possess functions including light absorption, charge production/separation, and catalysis for efficient O_2_ production from H_2_O through photosynthesis principles based on bioelectronics and chemical biology studies focused on natural plants' mechanisms [27,28]. Scholars have recently paid attention to thylakoids within the chloroplast matrix-flat sacs arranged parallel along its long axis surrounded by a single membrane [29,30]. The thylakoid membrane contains photosynthetic pigments and components of the electron transport chain, where the conversion of light energy into active chemical energy (photoreaction) occurs [[31], [32], [33]]. Thylakoids are not only the smallest units capable of photosynthesis and oxygen production, but they also possess efficient photodynamic and photothermal conversion capabilities due to their significant light-trapping ability in the visible/near-infrared region [34,35]. Therefore, compared with traditional small molecule photosensitizers, thylakoid membranes possess multiple advantages. On one hand, thylakoid membranes have highly efficient light energy capture and conversion capabilities [31,36]. In contrast, small molecule photosensitizers have limited light energy capture range and conversion efficiency. Moreover, they can cause phototoxic side effects due to accumulation in non-target tissues [37,38]. On the other hand, thylakoid membranes have good stability in organisms because plants have evolved a set of ROS detoxification systems (such as SOD, CAT and POD) to detoxify stress-induced ROS [39,40]. So, it can resist external environmental interference and changes. Small molecule photosensitizers are easily inactivated by environmental factors. Additionally, green plants are rich in thylakoid membranes, making large-scale acquisition feasible for future transformation research. This results in effective killing effects on tumor cells through photodynamic and photothermal therapies, opening up new avenues for cross-species biomaterial research.

In this study, a novel hybrid system was designed by incorporating tumor cell membranes with thylakoid membranes based on the advantages and disadvantages of the aforementioned treatment methods (Schematic 1). The biomimetic drug delivery system is assisted by tumor cell membrane proteins CD47, programmed cell death ligand-1, and β2-microglobulin to facilitate immune escape [[41], [42], [43]]. By utilizing the natural properties of cell membranes, such as targeted delivery to reduce toxic side effects of anti-proliferative drugs, along with enhanced production of reactive oxygen species through unique light capture performance from thylakoid' PDT part, efficient inhibition of tumor growth can be achieved.

**Schematic 1:**
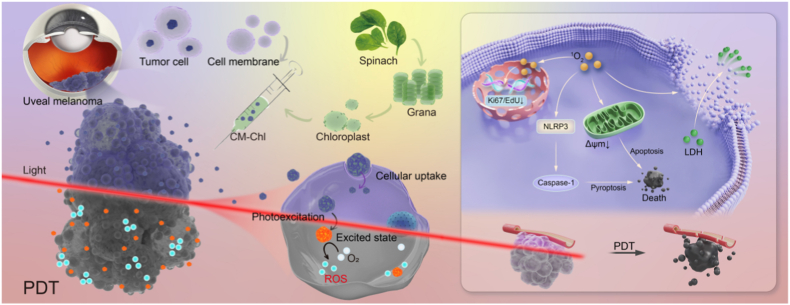
Homologous targeting of membrane-coated plant-derived thylakoids for photodynamic therapy of choroidal melanoma.

In specific experiments conducted for this study, fresh spinach was first isolated and purified. The successful extraction of thylakoids was confirmed through UV spectrum analysis, morphological observation, and SDS-PAGE analysis. Subsequently, homologous membranes from the choroidal melanoma cell line (OCM-1) were successfully collected using the ultrasonic cell fragmentation technique. The successful formation of membrane-thylakoid (CM-Thy) hybrids was confirmed through particle size potential measurement, UV spectroscopy analysis TEM imaging, and SDS-PAGE analysis. The CM exhibits homologous targeting properties. The uptake ability of CM was verified by a behavioral study of CM-Thy and OCM-1 cells. The biosafety of pure Thy was tested in OCM-1 and the possibility of Thy as a photosensitizer for PDT treatment was verified. In this process, a potential therapeutic strategy, which relies on white light to drive Thy production for enhancing tumor cell apoptosis, was discovered, and intensive exploration was carried out to elucidate its underlying mechanism of action. Within this system, Thy is capable of generating ^1^O_2_, and this specific ROS can induce a marked increase in intracellular ROS levels, subsequently initiating cellular lipid peroxidation responses and augmenting cell membrane permeability. Further mechanistic studies confirmed that the core functional pathway of this intervention therapy is characterized by dual effects of singlet oxygen. On one hand, it directly causes DNA oxidative damage and impairs cell proliferation potential, on the other hand, it activates the NLRP3 inflammasome pathway to mediate cell pyroptosis. These multiple biological effects work in synergy to ultimately induce apoptotic cell death. The multifunctional inhibitory effect of PDT therapy with CM-Thy was confirmed in experimental models of tumor-related processes (angiogenesis, vasculogenic mimicry, cell migration, etc.). In vivo experiments showed that the targeted CM-Thy could inhibit the proliferation of tumor cells and achieve the therapeutic effect of intraocular tumor volume reduction.

Therefore, in this study, a novel tumor membrane-thylakoid membrane hybrid was designed and synthesized, which can perform both targeted delivery and PDT. In doing so, the possibility of daily light source as a treatment modality was explored, and in the case of PDT, the potential is limitless. This concept of "take it from cancer, apply it to cancer" combined with cross-species biological therapy undoubtedly provides a new basis for the treatment of ocular tumors and creates new ideas for the subsequent development of milder and safer treatment methods, which has broad application prospects.

## 2 Materials and methods

### 2.1 Materials

Fresh spinach, gauze, and a kitchen juicer were purchased from a local supermarket. D-sorbitol, Magnesium chloride (MgCl_2_), Sodium ascorbate, Dimethyl sulfoxide (DMSO), Ethylenediaminetetraacetic acid (EDTA), Polyethyleneimine (PEI), Sucrose, and 1,3-Diphenylisobenzofuran (DPBF) were bought from Aladdin. Albumin was derived from sigma. Percoll cell isolation solution was purchased from Solebol. HEPES-KOH and MOPS-KOH were purchased from Yuchun Biology. Bovine serum albumin (BSA) was purchased from Sigma. The 0.22 μm Needle filter was purchased from JET BIOFIL. Yarnase Rainbow prestain protein Marker WJ103 was purchased from EpiZyme. Acetone Polycarbonate membranes (800、400、200、100 nm) were purchased from Avestin.

The cell counting kit-8 (CCK-8), Hoechst 33342, Calcein/PI Cell Viability/Cytotoxicity Assay Kit, 4% paraformaldehyde fix solution, Periodic Acid-Schiff Staining Kit (PAS), Singlet Oxygen Sensor Green Fluorescent Probe (SOSG), SOSG Assay Kit, lactate dehydrogenase (LDH) Release Assay Kit, Lipid Peroxidation Assay Kit with BDPY 581/591C11, BeyoClick™ Plus EdU Cell Proliferation Kit with AF488, Immunostaining Permeabilization Solution with Triton X-100, Primary Antibody Dilution Buffer, RIPA Lysis Buffer, Phenylmethanesulfonyl fluoride (PMSF), Antifade Mounting Medium with DAPI, enhanced BCA protein assay (BCA) kit, Membrane, and Cytosol Protein Extraction Kit, Coomassie Blue Fast Staining Solution, SDS-PAGE Sample Loading Buffer, TUNEL apoptosis assay kit, and 3,3′-dioctadecyloxacarbocyanine perchlorate (DiO) were purchased from Shanghai Biyuntian Biological Co., Ltd. The Mitochondrial Membrane Potential Detection Kit (JC-10) was purchased from ZETA LIFE Inc. Dulbecco's modified Eagle's medium (DMEM), DMEM/F12 (1:1), DMEM/F-12 containing L-glutamine, Fetal bovine serum (FBS), 0.05% trypsin-EDTA, penicillin-streptomycin solution, and other cell culture-related reagents were purchased from Gibco. Phosphate-buffered saline (PBS) was purchased from Boster Biological Technology. Matrigel 356234 and 356230 were purchased from Corning®.

P53 (ABT239R) Rabbit mAb, p53 (Phospho Ser46) (PT0903R) PT™ Rabbit mAb, Goat Anti Mouse IgG(H + L) (AbFluor 488), and Goat Anti-Mouse IgG(H + L) (AbFluor 594) were purchased from Immunoway Biotechnology Co., Ltd. Anti-NLRP3 antibody [EPR23094-1] was obtained from Abcam plc. IL-1 beta Polyclonal antibody and Caspase 1/P20 Polyclonal antibody were procured from Proteintech Group, Inc. Alpha/beta-Tubulin Antibody (#2148), Anti-mouse IgG, HRP-linked Antibody (#7076), and Anti-rabbit IgG, HRP-linked Antibody (#7074) were acquired from Cell Signaling Technology, Inc.

### 2.2 Cell culture and animals

The ocular choroidal melanoma-1 (OCM-1) cells were cultured in RPMI-1640-based complete medium. Human lens epithelial cells (HLECs) and retinal pigment epithelium cells (RPEs) were propagated in the DMEM/F-12-based complete medium. Human corneal epithelial cells (HCECs) were propagated in a DMEM/F-12-based complete medium containing L-glutamine. Human umbilical vein endothelial cells (HUVECs) were used for the experiment on tube formation and were incubated in a DMEM complete medium. Every complete culture medium was supplemented with 10% FBS and 1% penicillin-streptomycin, and the cells were incubated at 37 °C with a CO_2_ concentration of 5%. The medium was changed every 2 to 3 days. Three independent biological replicates were performed for each cell experiment, with each replicate containing 3-5 technical replicates.

Female BALB/c nude mice aged between 4 and 6 weeks old with a body weight of approximately 20 ± 2 g and New Zealand white rabbits weighing between 2.5 and 3 kg were obtained from Beijing WeitongLihua Experimental Animal Technical Co., Ltd.

### 2.3 Thylakoid preparation

The thylakoids were isolated from fresh spinach purchased at the local supermarket using an improved method [35,44]. In brief, the purchased spinach was stored in a 4 °C refrigerator away from light until used. Prior to use, the green leaves were washed, the roots removed, and roughly chopped. The leaves and pre-cooled buffer I (330 mM D-sorbitol, 50 mM HEPES-KOH, 5 mM MgCl_2_, 0.1% bovine serum albumin) were then added to a kitchen juicer for crushing. The resulting solution was filtered through gauze to obtain filtrate which was subsequently centrifuged at 3000×*g* for 10 min. After removing the supernatant, the precipitate was re-suspended with precooled buffer II (300 mM D-sorbitol, 50 mM HEPES-KOH, 5 mM MgCl_2_, 2 mM EDTA, and 10 mM sodium L-ascorbate). A Percoll solution with a permeability gradient of 80/40% was utilized (80%: 80% v/v Percoll, 10 mM sodium L-ascorbate, 300 mM sucrose, 66 mM MOPS-KOH. 40%: 40% v/v Percoll, 10 mM sodium L-ascorbate, 300 mM sucrose, 25 mM MOPS-KOH). It was diluted with buffer III (10 mM HEPES-KOH, 10 mM MgCl_2_, 10 mM sodium L-ascorbate) and stored at −80 °C for long-term storage purposes. 20 μL of the final solution mentioned above was mixed thoroughly and homogenized with a mixture of acetone solution containing 80% (v/v), followed by centrifugation at 3000×*g* for 3 min, and the supernatant was retained. The absorbance of the supernatant at 645 nm (A645) and 663 nm (A663) was determined using an ultraviolet spectrophotometer (UV-1780, Suzhou, China), and the concentration was calculated according to the following formula [[45], [46], [47]]:

C(mg/mL)=(8.02A663+20.2A645)/10.

### 2.4 Extraction of CM and preparation of CM-Thy

The OCM-1 cell membrane (CM) was extracted using a membrane protein extraction kit [48,49]. The extraction procedure was as follows: Firstly, 1x10^7^ cells were collected and washed three times with pre-cooled PBS. Subsequently, 1 mL of membrane protein extraction reagent A (including Phenylmethanesulfonyl fluoride) was added to fully suspend the cells. The resulting cell suspension was then subjected to ultrasonic decomposition (300 W, ultrasonic 3 s interval of 2 s, total ultrasonic time of 10 s) while being ice-bathed for 10 to 15 min. After ultrasonication, the cell suspension was centrifuged at 4 °C and 700×*g* for 10 min. The precipitate was discarded and the supernatant was collected. This supernatant underwent further centrifugation at 4 °C and 14000×*g* for 60 min before discarding the supernatant again. The resulting precipitate was represented the OCM-1 homologous cell membrane which was stored in a refrigerator at −80 °C. To obtain uniform-sized cell membranes, the aforementioned obtained OCM-1 cell membrane could be homogenized by extrusion using a liposome extruder (AVESTIN, LF-1, Canada) through polycarbonate membranes with pore sizes sequentially decreasing from 800 nm to 100 nm [50,51].

The CM was prepared to a protein concentration of 0.8-1 mg/mL (quantified by the BCA method), defined as one unit of CM. The Thy suspension was adjusted to a chlorophyll concentration of 1 mg/mL (determined by the acetone extraction method), defined as one unit of Thy. One unit OCM-1 cell membrane mixed with one unit thylakoids (1:1 w/w) also underwent extrusion using an extruder containing polycarbonate membranes with pore sizes of 800 nm, 400 nm, 200 nm, and 100 nm. The resultant solution after extrusion underwent centrifugation at 12000 rpm at 4 °C for 2 min followed by discarding the supernatant. Finally, 1 mL PBS solution was used to resuspend the precipitate, named CM-Thy. Re-calculation via the acetone extraction method showed that the chlorophyll content in one unit of CM-Thy accounted for 33.03 ± 6.32% of the initially added chlorophyll. CM-Thy will be diluted to the required concentration subsequently.

### 2.5 Characterization of Thy, CM, and CM-Thy

The size distribution and zeta potential of Thy, CM, and CM-Thy were analyzed using the dynamic light scattering method (DLS, Malvern Instrument Ltd, Malvern, UK) [34]. In order to maintain the uniformity of the solution, ultrasonic dispersion of the solution is performed prior to conducting the measurement. An ultraviolet spectrophotometer (UV-1780, Shimadzu, Japan) was utilized to measure the absorbance of each substance for lateral confirmation of the fusion between two membrane structures. Subsequently, transmission electron microscopy (TEM, FEI Talos F200, America) was employed to observe the surface morphology and three-dimensional structure of these substances [35]. For instance, in SDS-PAGE steps at 2.5%, proteins from OCM-1 cell protein along with CM-Thy and Thy were analyzed [52]. In simple terms, after quantification of the BCA protein extract, it was mixed thoroughly with 5 × sample buffer according to a specific ratio and heated at 100 °C for 10 min to denature the protein. The protein sample and marker were sequentially added into the sample wells, followed by the application of voltage until the completion of electrophoresis. After electrophoresis, the gel was gently washed with deionized water before pouring off excess liquid. Approximately 20 mL of Coomassie Blue Fast Staining Solution was added onto the gel surface for staining purposes. Following staining, decolorization was carried out by adding deionized water in a room temperature shaker for 2 h (with water changed every 10 min). Overnight decolorization with sufficient deionized water allowed for clearer visualization of protein bands on the gel surface which could be photographed and analyzed.

### 2.6 Metabolomics analysis

Metabolomics analysis was conducted on three types of samples (CM, Thy, and CM-Thy) using the Meiji Biological Platform. First, MS/MS analysis was performed on the included samples. After the MS/MS run was completed, the raw data were subjected to peak detection, extraction, alignment, and integration using Progenesis CD (Waters Corporation, Milford, USA). Meanwhile, substance annotation was accomplished using the HMDB database (http://www.hmdb.ca/), Metlin (https://metlin.scripps.edu/), and Meiji's in-house database.

To reduce errors in experiments and analysis, preprocessing was first carried out on the post-qualification data: features with >20% missing values in each group were removed, missing values were imputed with the minimum value of all samples, and the response intensity of sample mass spectral peaks was processed via sum normalization to obtain a normalized data matrix. Subsequently, variables with a relative standard deviation (RSD) > 30% in quality control samples were deleted, and log10 transformation was performed to get the final data matrix for subsequent analysis. For follow-up steps, statistical analyses such as principal component analysis (PCA) and orthogonal partial least squares-discriminant analysis (OPLS-DA) were conducted using the ropls package (Version 1.6.2) in R; metabolite annotation was implemented via the HMDB and KEGG databases (https://www.kegg.jp/kegg/pathway.html); and pathway enrichment analysis was performed using the scipy.stats package in Python.

### 2.7 Generation stability of ^1^O_2_

Before determining the ^1^O_2_, the storage stability of CM-Thy was first evaluated. The CM-Thy solution prepared by the aforementioned method was stored at 4 °C, and its particle size and zeta potential were measured continuously for seven days.

Subsequently, SOSG was employed to investigate the generation stability of ROS (SOSG exhibits high selectivity toward ^1^O_2_) [53]. To avoid the interference of the absorption peaks of CM and Thy with the characteristic peaks of SOSG, Thy was immobilized on the substrate surface via the electrostatic adsorption method reported in previous studies, thus anchoring the ROS-generating Thy at the bottom of the cuvette [54]. Briefly, the transparent substrate (polyethylene terephthalate, PET sheet) was first ultrasonically cleaned sequentially in anhydrous ethanol and deionized water, and then soaked overnight in a 3 mg/mL PEI solution. After rinsing with deionized water, the substrate was dried with nitrogen gas at room temperature to obtain a positively charged aminated surface. Subsequently, the treated substrate was soaked overnight in a pre-prepared Thy solution, followed by three rinses with PBS and drying with nitrogen gas [54].

The Thy-immobilized PET sheet was placed in a fluorescence cuvette, and 2 mL of 5 μM SOSG solution was added into the cuvette. The system was then irradiated with a 660 nm laser at a power density of 0.5 W/cm^2^ for 3 min. Immediately after irradiation, the fluorescence intensity of the mixture was measured using a fluorescence spectrophotometer. Measurements were taken every 5 min under dark conditions for a total duration of 30 min. Subsequently, the SOSG solution was removed, and 2 mL of fresh 5 μM aqueous SOSG solution was supplemented. The above irradiation and detection procedures were repeated, and this operation was performed three times in total. The fluorescence intensity changes of SOSG during the three cycles were calculated.

In addition, a new Thy-immobilized sheet was placed in a fluorescence cuvette following the same procedure described above. SOSG solution was added, and the system was irradiated with laser. The fluorescence spectra before irradiation and 10 min after irradiation were recorded as the control spectra at day 0. The cuvette was then stored in the dark. Over the subsequent 14 days, the SOSG solution was replaced at regular intervals, and the same Thy-immobilized sheet was irradiated repeatedly. Meanwhile, the fluorescence spectra of the SOSG solution in the cuvette were recorded. The fluorescence intensity changes of SOSG at different time points were compared to investigate the ^1^O_2_ generation stability [53].

On the basis of verifying that Thy is capable of ^1^O_2_ generation, another probe was used to investigate the ^1^O_2_ generation capacity of CM-Thy. DPBF functions as a highly efficient ^1^O_2_ quencher and rapidly reacts with ^1^O_2_ [[55], [56], [57]]. To evaluate the singlet oxygen generation ability of CM-Thy, the attenuation of the DPBF absorption peak at 415 nm was measured. Initially, DPBF was dissolved in DMSO to prepare a solution with a final concentration of 0.5 μM. Then, 2 mL of this prepared DPBF solution was transferred into a UV quartz cuvette for subsequent experiments. CM-Thy was mixed promptly with fresh DPBF and kept in darkness until further use. The UV absorption spectrum (330 nm-500 nm) of the mixture was recorded at different time intervals within 10 min by irradiating it with light at a wavelength of 660 nm and power density of 0.5 W/cm^2^. Additionally, under identical light treatment conditions (power density: 0.5 W/cm^2^; duration: 3 min), pure DPBF solution without CM-Thy (control group) was also subjected to illumination, and its absorbance value at regular intervals was measured alongside that of the CM-Thy/DPBF mixture at 415 nm for statistical analysis.

### 2.8 Research on targeting properties of homologous CM

To visualize cell membranes, DiO reagent (green fluorescence) was used on extracted cell membranes while DiO-CM was centrifuged at 12000×*g* for 2 min after being mixed with DiO, then incubated at 37 °C for 30 min before being resuspended with PBS and stored at 4 °C for future use [[58], [59], [60]].

Intraocular normal cells such as HCECs, HLECs, RPEs, and the tumor cells OCM-1 were inoculated into a 24-well plate with a density of 6 × 10^4^ per hole followed by serum-free medium starvation treatment after adhesion. The complete medium containing DiO-CM was added to each well. After removing the culture medium for 4 h, 4% PFA was added into each well fixed away from light for 10 min, and then the excess dye and fixing solution was cleaned using PBS. The cell slides containing attached cells were removed inverted onto a slide, and sealed with an antifade mounting medium with DAPI. Finally, the confocal laser scanning microscope (LSM880, Zeiss, Germany) was used to obtain fluorescence images which were quantitatively analyzed.

### 2.9 Observation of CM-Thy uptake behavior

In order to assess the uptake capacity of OCM-1 cells for CM-Thy, OCM-1 cells were seeded in 24-well plates. After starvation treatment, Thy and DiO-CM-Thy solutions were added to two separate groups. Following co-incubation for 4 h, fixation was performed using 4% PFA, followed by three washes with PBS. Subsequently, an antifade mounting medium with DAPI was added and the wells were sealed. Confocal laser scanning microscopy (LSM880, Zeiss, Germany) was used for local observation of the cells. The micrographs of DiO-CM and Thy at the same scale were compared.

### 2.10 The exploration of inducing anti-tumor effect under the daily light source

ROS accumulation naturally occurs in plant leaves under sunlight exposure [[61], [62], [63]]. Therefore, this part investigated whether Thy exhibits anti-tumor properties under low-intensity light sources as well. To verify this hypothesis, the first step was to examine its apoptosis-inducing behavior on tumor cells in vitro. OCM-1 cells were first seeded into 96-well plates at a density of 5 × 10^3^ cells/well and allowed to attach to the surface before adding different concentrations of Thy solutions (final concentrations were 2.5, 5, 10, 20, 40, 80, and 100 μg/mL). Each group included five parallel controls. After incubating for 24 h, the plate wells were irradiated with visible light (60 mW/cm^2^) for 1 h while avoiding light exposure served as the control group.

In order to qualitatively investigate the survival status of the cells, the cells in the plates were stained using the Calcein/PI cell viability/cytotoxicity assay kit [64]. The procedure involved removing the old medium and adding 100 μL of the prepared Calcein AM/PI detection working solution (Calcein AM: PI: detection buffer = 1 μL: 1 μL: 1 mL) into each well, followed by incubation for 30 min away from light. Calcein AM can be enzymatically converted by esterase within cells to produce calcein, which exhibits green fluorescence under microscope excitation at 490 nm. The nuclear dye PI can only penetrate membrane-damaged regions of dead cells and bind to DNA double helix structures within them, emitting red fluorescence upon excitation. Previous studies have also shown that Thy (due to the presence of Chlorophyll) spontaneously fluoresces in red. Therefore, after discarding the Calcein AM/PI detection solution, Hoechst33342 was used for nuclear staining, and fluorescence images were captured using an inverted fluorescence microscope (DMi8, Leica, Germany) after a further incubation period of 10 min [65].

Additionally, the plates prepared in a similar manner were utilized for qualitative assessment of cell viability using CCK-8 assay [54,66]. After exposure to visible or dark light treatment, a fresh medium containing CCK-8 reagent was added to each well and incubated at 37 °C for 2 h. The absorbance was measured at 450 nm using a multi-functional ultraviolet enzyme labeling instrument (SpectraMax 190, USA) to compare the impact of Thy on the tumor cells.

### 2.11 Study on antitumor properties under 660 nm laser

The antitumor photodynamic therapy commonly utilizes a 660 nm laser, which is more frequently employed in this field [66,67]. In addition, the 660 nm laser exhibits extremely low toxicity toward normal cells and is not readily absorbed in large quantities by major components of biological tissues such as hemoglobin and water. While activating the photosensitizer at the target site, it can reduce photothermal and oxidative stress damage to the surrounding normal tissues, thereby improving the safety and tolerability of the therapy [68]. In order to enhance treatment efficiency, we also investigated the antitumor effect of Thy induced by a visible range 660 nm laser (IR laser, Yuanming, China). The cells were seeded following the same procedure as described before and subsequently exposed to vertical irradiation from a 660 nm laser after cell adhesion. The gradient concentration of Thy was set at 1, 10, and 20 μg/mL, while the irradiation power was fixed at either 0.5 W/cm^2^ or 1 W/cm^2^ for a duration of 3 min. As control groups, the plates away from light were utilized. Qualitative and quantitative analysis of the cells was conducted using the same method.

### 2.12 Characteristics of CM-Thy-induced apoptosis

#### 2.12.1 Analysis of intracellular ROS release capacity

In the context of PDT treatment for choroidal melanoma, intracellular ROS content is pivotal. The ROS release capacity of OCM-1 cells before and after CM encapsulation was analyzed under various concentrations of screened Thy. To measure ROS production, OCM-1 cells were seeded into a 96-well plate at a density of 5 × 10^3^ cells/well. PBS, Thy, and CM-Thy solution were added and incubated for 4 h followed by removal of the drug solution. DCFH-DA reagent was then added to co-incubate with the cells for 20 min before washing three times with serum-free medium [66]. Irradiation at intensities of 0.5 W/cm^2^ and 1 W/cm^2^ was performed for 3 min each time using an inverted phase contrast fluorescence microscope (DMi8, Leica, Germany).

#### 2.12.2 Detection of LDH release behavior

Experimental groups included a positive control group, blank group (negative control), CM-Thy group, simple light group, and CM-Thy light group as before using the plate seeding method. After co-incubation for 24 h, the original medium was replaced with a serum-free medium following suctioning out and washed with PBS buffer. The positive control group received the addition of 10% LDH release reagent based on the volume of the original medium and was repeatedly mixed [69]. After replacing the original medium, the samples were irradiated with a laser at a power of 0.5 W/cm^2^ for 3min.

Next, the detection of LDH release was performed by preparing an LDH working solution and diluting INT (10X) to INT (1X). The LDH working solution (lactic acid solution: INT (1X): enzyme solution = 20 μL: 20 μL: 20 μL) was then prepared. After co-incubation for 24 h, the plates were centrifuged at a speed of 400 rpm for 5 min, and 120 μL of supernatant from each pore was added to new pore plates. Each well was then supplemented with 60 μL of LDH working liquid, thoroughly mixed by blowing and incubated in a light-free shaker at room temperature for 30 min. The absorbance value at 490 nm was measured using a plate reader to calculate the LDH release rate (with the positive control group set as 100% and subtracting the absorbance value of each group from that of the background blank control well).

#### 2.12.3 Detection of ^1^O_2_

The SOSG Kit was used to detect intracellular ^1^O_2_ in cells treated with CM-Thy. Three experimental groups were set up: the control group, PBS group, and CM-Thy group (with the Thy concentration maintained at 20 μg/mL). The cells in the latter two groups were subjected to two irradiation regimens, respectively: visible light irradiation for 1 h, and 660 nm laser irradiation (0.5 W/cm^2^) for 3 min.

First, 100 μg of SOSG was dissolved in 33 μL of methanol to prepare a stock solution with a concentration of approximately 5 mM. Subsequently, the SOSG working solution was prepared fresh under dark conditions as required, containing the corresponding amount of SOSG and Hoechst33342. OCM-1 cells were seeded in 96-well plates. The control group received no additional treatment, while the cells in the other groups were subjected to the aforementioned interventions. Then, 100 μL of the SOSG working solution was added to each well, and the plates were incubated at 37 °C in the dark for 15 min. After incubation, images of each group were captured under a 20 × fluorescence microscope, and quantitative analysis was performed on the green fluorescence signals (derived from SOSG endoperoxide, Ex/Em = 504/525 nm) in the cells.

#### 2.12.4 Detection of intracellular lipid peroxidation level

BDPY 581/591C11 was employed to detect intracellular lipid peroxidation [70]. The staining working solution (2 μM) was prepared by mixing 1 μL of 2 mM BDPY 581/591C11 with 1 mL of PBS. The experiment was divided into the same three groups: control group, PBS group, and CM-Thy group (Thy concentration: 20 μg/mL). The cells in the PBS and CM-Thy groups were irradiated with visible light for 1 h or 660 nm laser for 3 min, respectively.

After intervention, the culture medium was aspirated from the cells, and 1 mL of the prepared staining working solution was added to each well. The cells were incubated at 37 °C for 15 min. Following incubation, the supernatant was discarded, and the cells were washed twice with PBS. Then, 100 μL of PBS was added to each well, and the cells were observed under a 20 × fluorescence microscope. The cells exhibiting green fluorescence (corresponding to the oxidized form, Ex/Em = 488/510 nm) were imaged, and the lipid peroxidation level was evaluated by analyzing the intensity of the green fluorescence signals [71].

#### 2.12.5 Detection of mitochondrial membrane potential

JC-10 was used for the detection of mitochondrial membrane potential [72]. First, 50 μL of 200 × JC-10 stock solution was added to 8 mL of ultrapure water and dissolved by vigorous shaking. Subsequently, 2 mL of 5 × staining buffer was added and mixed thoroughly to prepare the JC-10 staining working solution. The experimental grouping was consistent with the aforementioned protocols. Adherent cells cultured in 96-well plates were used for the assay: the culture medium was aspirated, and the cells were washed once with PBS. Then, 100 μL of cell culture medium and 100 μL of JC-10 staining working solution were added to each well and mixed well. The cells were incubated at 37 °C for 20 min. During the incubation period, 1 × staining buffer was prepared by mixing 1 mL of 5 × staining buffer with 4 mL of distilled water, followed by ice-bathing.

After incubation, the supernatant was removed, and the cells were washed twice with 1 × staining buffer. Finally, 200 μL of cell culture medium was added to each well, and the cells were observed under a 20 × fluorescence microscope. Two fluorescence parameters were set: green fluorescence (corresponding to JC-10 monomers, Ex/Em = 490/530 nm) and red fluorescence (corresponding to JC-10 aggregates, Ex/Em = 525/590 nm). Images were captured, and the changes in mitochondrial membrane potential were quantified by analyzing the green fluorescence intensity [73].

### 2.13 Study on the mechanism of CM-Thy-induced apoptosis

#### 2.13.1 Evaluation of proliferative capacity and pyroptosis pattern of tumor cells

The EdU assay was used to detect cell proliferation, with the grouping and intervention methods consistent with the aforementioned protocols [74]. First, 10 mM EdU was diluted with cell culture medium at a ratio of 1:500 to prepare a 20 μM 2 × EdU working solution [75]. Cells cultured overnight in 96-well plates were supplemented with an equal volume of 2 × EdU working solution to achieve a final concentration of 10 μM. After incubation at 37 °C for 2 h, the culture medium was aspirated, and 100 μL of fixative solution was added for fixation for 15 min. The cells were then washed three times with PBS, followed by incubation with an appropriate volume of permeabilization solution at room temperature for 15 min and two additional washes. Subsequently, the Click reaction solution was prepared at a reduced ratio (with a 50 μL reaction system per well). 50 μL of the reaction solution was added to each well and incubated at room temperature for 30 min in the dark. After removing the reaction solution, the cells were washed three times with washing solution. Separately, a 1 × Hoechst33342 staining solution was prepared, and 100 μL of this staining solution was added to each well for incubation at room temperature for 10 min in the dark, followed by three washes. Finally, the cells were observed under a 20 × fluorescence microscope. The excitation/emission wavelengths were set as 495/519 nm for Picolyl Azide 488 and 346/460 nm for Hoechst33342. Fluorescent images were captured for quantitative analysis of fluorescence signals related to cell proliferation.

Immunofluorescence assay was employed to investigate the overall proliferative capacity of cells and the initiation of apoptosis (phosphorylation of p53 protein) [76]. The cell seeding and grouping methods were the same as previously described. The detailed procedures were as follows: after aspirating the culture medium from cells in each group, the cells were washed three times with PBS. An appropriate volume of paraformaldehyde solution was added for fixation for 10 min, followed by three washes with PBS on a shaker (5 min per wash). The cells were then incubated with permeabilization solution at room temperature for 30 min, and washed three more times with PBS on a shaker (5 min per wash). Blocking was performed with 5% BSA blocking solution for 1.5 h. Subsequently, primary antibodies against Ki67, p-P53, and p53 were diluted with antibody dilution buffer at a ratio of 1:500, respectively, and the diluted primary antibodies were added to the corresponding wells for overnight incubation. On the following day, after washing, the cells were incubated with species-matched secondary antibodies diluted at a ratio of 1:1000. After adding fluorescence anti-fade mounting medium containing DAPI for mounting, fluorescent images were acquired under a confocal laser scanning microscope, and the fluorescence signals of target proteins were subjected to quantitative analysis.

In addition, immunofluorescence assay was also used to explore the ^1^O_2_-driven pyroptosis behavior associated with the NLRP3 inflammasome, following the same protocol described above [77,78]. The primary antibodies used were anti-NLRP3, anti-Caspase-1, and anti-IL-1β.

#### 2.13.2 Western Blot analysis

Western Blot was used to detect the expression levels of cell proliferation-related proteins Ki67, p-p53, p53, and pyroptosis-related proteins (NLRP3, Caspase-1, IL-1β), with tubulin as the internal reference protein [79]. The cell grouping and intervention methods were consistent with the aforementioned protocols. The detailed experimental procedures were as follows: cells in each group were washed three times with pre-chilled PBS, and then lysed with strong RIPA lysis buffer containing PMSF. The cells were scraped off the plate until the lysate became clear, and then transferred to centrifuge tubes. The lysates were sonicated on ice and lysed for 20 min, followed by centrifugation at 14,000 rpm and 4 °C for 5 min. The supernatant was collected as the total protein extract. The protein concentration of the samples was determined using the BCA assay. Protein samples were denatured by boiling at 100 °C for 10 min, and then subjected to SDS-PAGE electrophoresis, membrane transfer, and blocking. After discarding the blocking solution, the membranes were incubated overnight at 4 °C with diluted primary antibodies against Ki67, p-p53, p53, NLRP3, Caspase-1, IL-1β, and tubulin (all diluted at a ratio of 1:1000). On the next day, after washing the membranes, they were incubated with diluted secondary antibodies (1:2000) at room temperature for 1 h. After another round of washing, ECL chemiluminescence reagent was added dropwise onto the membrane surface, and the membranes were exposed in a darkroom for several seconds. Finally, the gray values of each protein band were analyzed.

### 2.14 Influence of PDT effect of CM-Thy on the tumorigenesis mechanism

#### 2.14.1 Establishment of vasculogenic mimicry and evaluation of the inhibitory effect

OCM-1 cells were cultured under normal oxygen and hypoxia conditions, while continuously observing their growth state and formation of tubular structures [80,81]. The experimental procedure involves implanting OCM-1 cells in a density of 4 × 10^4^ cells/well in a 24-well plate. Once attached to the surface, they were cultured separately in hypoxic conditions (1% oxygen) or normoxic incubators. The medium was changed every 2-3 days with ongoing monitoring. Cell morphology was recorded using an inverted microscope (Axio Observer3, ZEISS, Germany) under bright field illumination.

In addition to observing cell morphology under bright field illumination, Hoechst33342 staining was used to visualize nuclear morphology more clearly during cell growth. PAS staining was also employed to observe tubular structure formation during the growth process [82,83]. The procedure involves immobilizing cultured cells with 70% ethanol for 10 min followed by oxidation with an iodic acid solution in darkness and washing with PBS for 5 min. Subsequently, Schiff reagents were applied to stain the samples at 37 °C for 30 min. After washing each well was stained with hematoxylin for 30s and the excess color was cleaned with PBS, and then photographed under a microscope (Axio Observer3, ZEISS, Germany). Subsequently, quantitative analysis was performed on the fluorescence intensity of Hoechst and angiogenesis-related indicators.

#### 2.14.2 Angiogenesis experiment and inhibitory effect evaluation

Tumor development involves the secretion of high levels of pro-angiogenic factors that promote the angiogenesis process. To examine the effect of PDT therapy with CM-Thy on this phenomenon, human umbilical vein endothelial cells (HUVECs) were utilized as endothelial cell models for simulating angiogenesis on low-growth-factor-containing Matrigel gel [84,85]. The experimental procedure was conducted as follows: 50 μL of Matrigel gel was added to each well and incubated for 30 min. Subsequently, 1.5 × 10^4^ cells were seeded in each well along with 100 μL of conditioned medium (OCM-1 cell culture supernatant filtered at 220 nm). Continuous observation was carried out at different time points (4 h, 6 h, 8 h, and 12 h) with corresponding photographs taken. For the treatment groups, OCM-1 cells were subjected to PDT according to the method described as before. The tumor supernatant after treatment was then used to culture HUVECs for subsequent comparison of angiogenesis status with the control group. After recording the bright-field images, statistical analysis was performed on the angiogenesis-related indicators.

#### 2.14.3 Exploring the ability to inhibit the migration of tumor cells

In order to investigate the PDT therapy of CM-Thy on OCM-1 cell migration, a cell scratch experiment was conducted [86,87]. The cells were initially seeded in 24-well plates with 500 μL of complete media at a density of 4 × 10^4^ cells/well. After 24 h of incubation, a sterile gun tip was used to create a cell scratch, followed by the removal of free cells using PBS. Subsequently, the cells were divided into four groups (same as before). Following the addition of CM-Thy and illumination, continuous observation and photography were performed for 84 h on the same area of cells at different times. The areas covered by scratched cells were then quantified to calculate cell mobility.

#### 2.14.4 Evaluation of 3D tumor generation and inhibition effect

To assess tumor size formed by choroidal melanoma cells, a three-dimensional tumor model was cultured using Matrigel gel in a 96-well plate [[88], [89], [90]]. Solidification was achieved by adding 50 μL/well of Matrigel and incubating at 37 °C for 30 min. OCM-1 cells were subsequently inoculated into Matrigel-precoated wells at a density of 2 × 10^4^/well along without other treatment as a control group. PDT treatment described above was applied to choroidal melanoma cells and tumor formation was observed over the next 36 h. The diameters of tumor spheroids in each group were measured and statistically analyzed to evaluate the tumor-killing effect. To visualize tumor mass formation more clearly, Calcein AM/PI/Hoechst staining was performed on tumor cells at the endpoint (36h), and images from each fluorescence channel were captured under an inverted fluorescence microscope (ZEISS Axio Observer3, Germany). The green fluorescence intensity was statistically analyzed to evaluate the viability of tumor cells.

### 2.15 Establishment of choroidal melanoma model in vivo and the effect of intraocular treatment

The animal experiments were approved by the Committee and Laboratory Animal Center of Eye Hospital, Wenzhou Medical University. The ethical approval Number was YSG24061404. Mice without congenital eye disease were selected for further experimentation. A subretinal injection of a cell suspension containing 2 × 10^5^ OCM-1 cells in a volume of 2 μL was administered into the right eye of each mouse [91,92]. According to reports, the success rate of tumor formation using this modeling method can be as high as 100%, with successful tumor engraftment achieved within two weeks after injection [93]. On day three post-injection, mice were randomly divided into four groups: PBS group, light group, Thy group, and PDT group, and three animals were assigned to each group. Intravitreal injections of PBS or CM-Thy were given into their right eyes while light treatment every two days was performed on the light and PDT groups. The injection volume of each group is mentioned as 2 μL, and the concentration of Thy is maintained at 200 μg/mL. The left eye served as the control group throughout the experiment.

Nude mice were observed daily for leukocoria and exophthalmos post-injection. After two weeks of treatment, surgical slit lamp examination and photography were conducted on euthanized nude mice's eyeballs which were subsequently separated and weighed before being fixed using the fixative solution. Fixed eyeballs underwent sagittal plane dissection followed by microscopic observation of internal tumors. Subsequently, quantitative statistics were performed on the tumor area and ocular volume in the section pictures of each group to evaluate the tumor reduction effects of each group.

The fixed tissues were subjected to pathological sections with a thickness set at 5 μm and stained with hematoxylin-eosin (H&E) staining. After the staining was completed, the slides were sealed with neutral resin, and then dried on the neutral resin and photographed on a 3D slide scanner (3D Pannoramic, Belgium) to evaluate the size of the intraocular tumor tissue and the growth of tumor cells.

TUNEL staining was performed on the sections. In brief, cells were fixed with 4% PFA for 30 min and washed twice with PBS for 10 min each time. Subsequently, they were incubated in PBS containing 0.5% Triton X-100 at room temperature for 5 min before being treated with a prepared TUNEL staining solution (consisting of 5 μL TdT enzyme, 45 μL fluorescent labeling solution, and TUNEL detection solution) and incubated at 37 °C for an hour in the dark. After washing three times with PBS, an antifade mounting medium with DAPI was added to the slice which was then observed under a fluorescence microscope (LSM880, Zeiss, Germany) after being sealed with cover glass.

During the aforementioned experiments, synchronous observation was conducted on the ocular surface damage and tissue morphology of nude mice in the light-irradiated group. In addition, to further evaluate the safety of light irradiation as a therapeutic modality, another animal model (rabbits) was used to assess the functional changes in the retina after light irradiation treatment [94].

After three rounds of laser irradiation, the rabbits were placed in a dark room for 4 h of dark adaptation. After the rabbits were fully anesthetized, corneal electrodes, subcutaneous electrodes, and ground electrodes were connected sequentially. Subsequently, dark adaptation tests at 0.01 Hz and 3.0 Hz were performed in turn. Upon completion of the dark adaptation tests, the rabbits underwent 10 min of light adaptation, followed by light adaptation tests at 3.0 Hz and Flicker 30 Hz tests. Finally, statistical analysis was performed on the electroretinogram ERG waveforms and peak values under each test mode.

### 2.16 Statistical analysis

Data were expressed as mean ± standard error of the mean. Group differences were analyzed using one-way analysis of variance (one-way ANOVA), with statistical significance set at P < 0.05 (P < 0.05, P < 0.01, ∗P < 0.001).

## 3 Results and discussion

### 3.1 Component analysis of Thy and CM

**Fig. 1:**
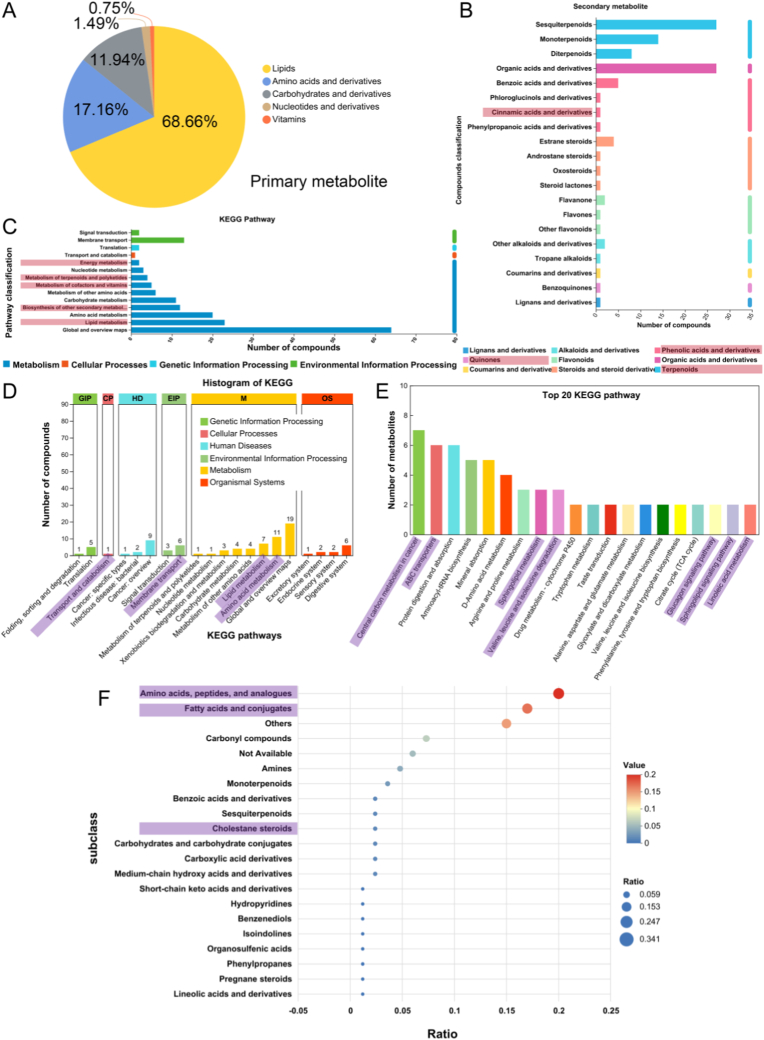
**Component analysis of Thy and CM.** A, B: Plant Metabolite Analysis of Thy; A: Primary Metabolite Classification, B: Secondary Metabolite Classification. C: Thy-Related KEGG Functional Pathways. D: CM-Related KEGG Functional Pathways. E: Top 20 CM-Related KEGG Functional Pathways. F: CM-Related Compound Classification from Human Metabolome Database (HMDB).

Fig. 1 focuses on the analysis of plant metabolites in Thy and CM samples. For the classification of primary metabolites (Fig. 1A), Thy contains 68.66% lipids (cell membrane components), 17.16% amino acids and their derivatives (protein precursors), while chlorophyll belongs to the category of lipids and their derivatives. Regarding the classification of secondary metabolites (Fig. 1B), terpenoids and organic acid derivatives are the most abundant. Notably, the metabolites related to thylakoids in spinach are terpenoids (directly involved in ROS production), quinones (involved in superoxide anion generation), and phenolic acids and their derivatives.

Fig. 1C shows the enrichment analysis of KEGG pathways related to Thy. The pathways associated with thylakoid- and chlorophyll-mediated ROS production include energy metabolism, terpenoid and polyketide metabolism, cofactor and vitamin metabolism, lipid metabolism, and biosynthesis of other secondary metabolites.

Fig. 1D presents the enrichment analysis of KEGG pathways related to CM. Pathways associated with CM include signal transduction, membrane transport, lipid metabolism, and amino acid metabolism. Additionally, "transport and catabolism" under the "Cellular Processes" category involves membrane-related substance transport, which indirectly participates in the metabolic regulation and functional control of tumor cells. Among the top 20 KEGG-related pathways (Fig. 1E), pathways involved in tumor metabolic reprogramming (central carbon metabolism in cancer, branched-chain amino acid metabolism), tumor cell membrane substance transport and drug resistance (ABC transporters), tumor cell membrane lipid metabolism and signaling pathways (sphingolipid metabolism, linoleic acid metabolism), and tumor cell membrane signaling pathways (glucagon signaling pathway, sphingolipid signaling pathway) directly or indirectly contribute to the occurrence and development of choroidal melanoma. Furthermore, some molecules in these pathways (e.g., ABC transporters, sphingolipid metabolic enzymes) can serve as potential targets for tumor-targeted therapy.

When searching for HMDB database-related pathways for CM (of animal origin) (e.g., Fig. 1F), it can be observed that metabolic subclasses of CM components—including amino acids, peptides and analogs, fatty acids and conjugates, and cholestane steroids—are closely associated with the occurrence, development, and targeted therapy of choroidal melanoma by regulating the composition and signal transduction of tumor cell membranes or acting as metabolic targets.

### 3.2 Preparation and characterization of CM-Thy

Preliminary observation of spinach leaf extracts showed that they exhibited chloroplast morphology under a light microscope (Fig. S1) and could emit spontaneous red fluorescence due to the presence of chlorophyll (Fig. S2). After ultrasonic treatment and liposome extrusion, the internal chlorophyll-rich thylakoids were released (Fig. S3).

**Fig. 2:**
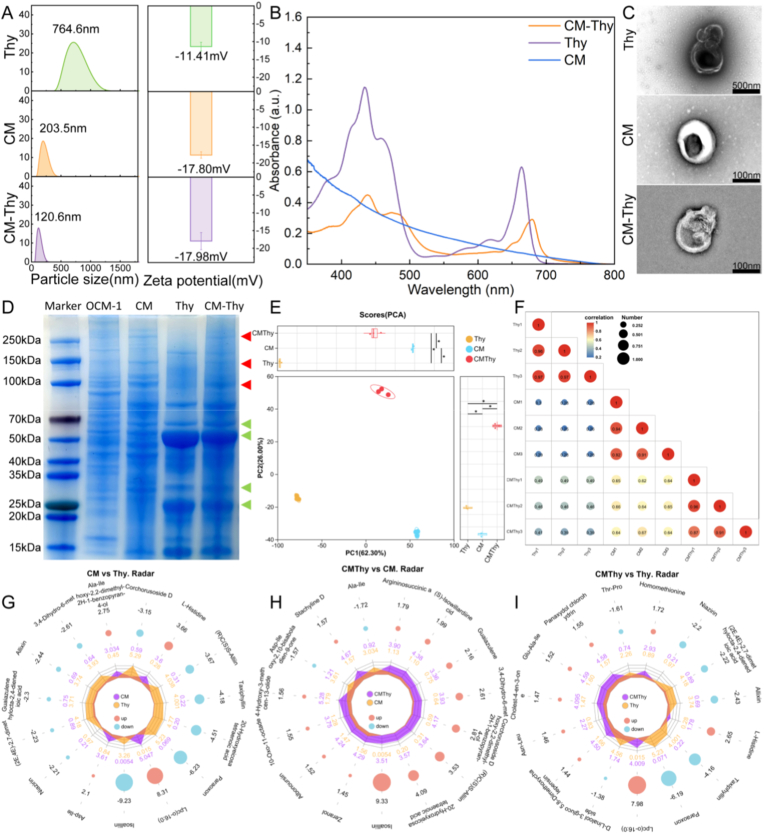
**Preparation and characterization of CM-Thy.** A: Particle size distribution and potential of Thy, CM, and CM-Thy. B: UV absorption images of Thy, CM, and CM-Thy. C: Transmission electron microscope images of Thy, CM, and CM-Thy. D: Coomassie Brilliant Blue of Thy, CM, and CM-Thy surface membrane protein compared with OCM-1 cells. Red arrows: marker proteins of CM, Green arrows: marker proteins of Thy. E: PCA Analysis Plot of Samples. F: Sample Correlation Analysis of Three Sample Types (CM, Thy, and CM-Thy). G-I: Statistical Analysis Plot of Differential Metabolites Among Three Sample Types (CM, Thy, and CM-Thy); G: Comparison Between CM and Thy, H: Comparison Between CM-Thy and Thy, I: Comparison Between CM-Thy and CM.

Characterization of drug-carrying nanoparticles in cell membranes includes evaluation of their physicochemical and biological properties, and the successful coating of the cell membrane depends on the size, surface charge, and protein composition of the prepared substance [95,96]. The extracted thylakoid size in Fig. 2A is measured to be 764.6 nm, with a membrane potential of −11.41 mV. After homogenization, the particle size of CM is reduced to 203.5 nm, while the potential becomes −17.80 mV. Mixing and extrusion of the two membrane structures result in a smaller size (120.6 nm) for CM-Thy, which facilitates subsequent uptake by tumor cells. It should be noted that there is no significant change in zeta potential, possibly due to the large proportion of CM that does not cause a substantial shift in potential value. In Fig. 2B, Thy exhibited strong absorption peaks in the regions of both 400-550 nm and 640-700 nm due to its chlorophyll components (ensured its PDT capability) [97,98].CM does not exhibit any distinct characteristic absorption peak compared to the standard curve of Thy. However, the characteristic absorption peak of CM-Thy shows a redshift behavior. The image of TEM in Fig. 2C reveals irregular morphology and rough surface for Thy alone, while CM forms vesicular structures through the extrusion process. However, when mixed as CM-Thy, both surface and interior become roughened compared to their states. From the large-scale images, it can be seen that the prepared CM-Thy is relatively evenly distributed (Fig. S4). The dimensions observed under electron microscopy align with the DLS results presented in Fig. 2A. It is worth highlighting that the sizes obtained from the two measurements are different from the results of microscopic examination in Figs. S1 and S2. This discrepancy might be attributed to the breakage of the intact chloroplast membrane and the leakage of internal thylakoids during the ultrasonic dispersion that was carried out before the measurement to make the solution homogeneous. This speculation has also been verified by the research of Scholar Chen [35]. Fig. 2D demonstrates the qualitative analysis of three substances along with proteins from OCM-1 cells. Both CM and CM-Thy show the presence of proteins at around 250 kDa, 150 kDa and 100 kDa (indicated by red arrows), whereas only CM-Thy contains characteristic proteins markers specific to Thy (marked by green arrows), particularly LHCPs which constitute a significant portion (accounted for the majority indicating successful extraction of chloroplasts as well as the release of internal thylakoids due to rupture of the restrictive envelope) [99].

Similarly, immunofluorescence was used to conduct a visual analysis of the cell membrane proteins related to cell membrane surface adhesion. It was confirmed in Fig. S5 that the CM contains the presence of membrane proteins such as E-Cadherin, ZO-1, EpCAM, CD81, and FN. Meanwhile, it was also verified that organelles such as α-SMA (a cytoskeletal protein), the cell nucleus, mitochondria, and lysosomes had been remove.

Fig. 2E presents the principal component analysis (PCA) of the samples, which allows for a preliminary understanding of the overall metabolic differences between sample groups and the degree of variation among samples within the same group. The PCA results show that the first principal component (PC1) explains 62.30% of the total variance, while the second principal component (PC2) explains 26.00 %. The samples are well-clustered, indicating good quality of biological analysis and data reliability. Meanwhile, this clustering suggests that the separation between groups is attributed to differential variables among groups rather than variations introduced during the analysis process.

In the sample correlation analysis shown in Fig. 2F, the correlation between CM-Thy and Thy, as well as between CM-Thy and CM, is significantly higher than that between Thy and CM. This may imply the hybrid behavior of CM-Thy, which enriches the biological information of the two individual substances (CM and Thy).

Fig. 2G–I further presents the statistical analysis of differential metabolites among the three sample types (CM, Thy, and CM-Thy). Fig. 2G shows the comparison between CM and Thy: upregulated substances such as L-histidine, dipeptides, and lysophosphatidylcholine are related to tumor cell membranes; downregulated substances including Corchorusoside D, Taxiphyllin, 20-hydroxyeicosatetraenoic acid, and sulfur-containing as well as benzopyran-based antioxidants are associated with thylakoids. Fig. 2H and I displays the comparisons between CM-Thy and the individual components (CM and Thy). In Fig. 2H, upregulated substances such as L-Histidine, LPC (o-16:0), Cholest-4-en-3-one, Asn-Leu, Glu-Ala-Ile, and homomethionine are related to tumor targeting. Among these upregulated substances, 20-Hydroxyeicosatetraenoic acid is a derivative of polyunsaturated fatty acids (PUFAs), ^1^O_2_ generated during photosynthesis attacks PUFAs and triggers lipid peroxidation, while other upregulated substances include thylakoid-related metabolites.

Notably, functional annotation and pathway mapping of differential metabolites reveal that CM-Thy samples not only retain some metabolic characteristics derived from CM and Thy but also exhibit new metabolic behaviors. This phenomenon may be attributed to the interaction between the two components (CM and Thy), which activates or inhibits certain metabolic pathways. For example, changes in key pathways such as sphingolipid metabolism and linoleic acid metabolism provide a new perspective for understanding the dynamic changes in tumor cell membranes. In addition, the abnormal activation of branched-chain amino acid metabolism and the glucagon signaling pathway also implies the potential role of CM-Thy in energy metabolism and signal transduction.d through the method of gradient density centrifugation during the extraction process (Fig. S6). These findings confirm the successful preparation of CM-Thy.

### 3.3 Determination of ^1^O_2_ generation stability of CM-Thy

**Fig. 3:**
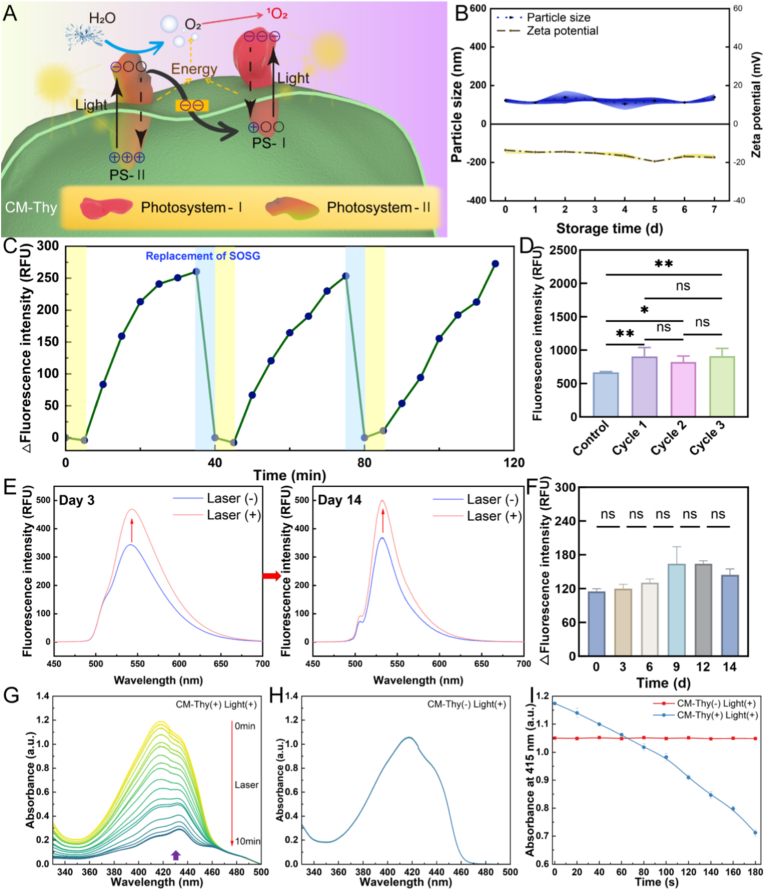
**ROS generation stability of CM-Thy. A: Mechanism of electron transfer and ^1^O_2_ generation in the CM-Thy hybrid membrane complex under light irradiation.** B: Changes in particle size and zeta potential of CM-Thy over seven consecutive days (n = 3). C: Curve of SOSG solution fluorescence intensity over time after irradiation. D: Fluorescence intensity of the solution at the end of each cycle in Figure C (n = 3). E: ^1^O_2_ capture and release capacity of thy after 3 and 14 days of storage in SOSG solution. F: Change in fluorescence intensity of SOSG solution after thy irradiation within 14 days (n = 3). G: Ultraviolet absorption spectra of DPBF in the CM-Thy/DPBF mixture after laser irradiation at different time points over a total duration of 10 min using a laser source at 660 nm. H: Ultraviolet absorption spectra depicting changes in DPBF absorbance over time when exposed solely to light without any additional components. I: Comparison between the absorbance values obtained from the CM-Thy/DPBF mixture and simple DPBF solution under varying durations of laser irradiation. All measurements were conducted for an illumination period lasting 3 min.

The results in Fig. 2H indicated that CM-Thy was involved in ^1^O_2_ generation, while Fig. 3A intuitively illustrated the light-driven synergistic process of photosystem II (PS-II) and photosystem I (PS-I) in the CM-Thy system, with its mechanism consistent with the functional logic of the Z-scheme structure [100,101]. Upon light excitation, PS-II underwent charge separation (the “+” and “−” symbols at PS-II in the figure correspond to holes and electrons, respectively). The holes with high oxidation potential could oxidize water molecules to release oxygen, a process indicated by the blue arrows in the figure. Meanwhile, the excited electrons in PS-II were transferred to PS-I through the electron transport chain on the thylakoid membrane (CM-Thy) (schematicized by black arrows and energy transfer markers), achieving efficient recombination of electrons with the holes in PS-I [102]. PS-I could also be excited by light. Combining the classical photosensitive properties of chlorophyll in PS-II and PS-I, the O_2_ generated by the system itself could be further converted into ^1^O_2_ via an energy conversion pathway (corresponding to the red arrows) [102,103]. The entire process clearly demonstrated the sequential reactions of water splitting for oxygen production, electron transfer and singlet oxygen generation in this system, directly verifying the synergistic characteristics of spatiotemporally synchronized oxygen self-supply and reactive oxygen species generation in the PS-II/PS-I system.

Prior to stable ^1^O_2_ generation, the stability of CM-Thy itself was crucial. The results in Fig. 3B showed that the average particle size of CM-Thy was stably maintained at approximately 120 nm and the zeta potential at −16 mV within 7 days of storage. This confirmed that CM-Thy exhibited uniform particle size and stable surface charge during storage, enabling long-term preservation of structural integrity.

Subsequently, SOSG, a specific indicator for ^1^O_2_, was used as a probe to characterize the laser-triggered ^1^O_2_ capture-release performance of the thylakoid components in CM-Thy [104]. The results in Fig. 3C revealed that the change in fluorescence intensity showed a cyclic upward trend with incubation time (0-120 min). After the SOSG replacement operation at 40 min, the fluorescence intensity fluctuated slightly but then resumed its upward trend, suggesting that the thylakoid components could achieve sustained ^1^O_2_ capture and release under near-infrared irradiation. In Fig. 3D, the fluorescence intensity of the control group was significantly lower than that of the 1st cycle group, while no significant difference was observed among the 1st, 2nd, and 3rd cycle groups. This indicated that the near-infrared-triggered ^1^O_2_ capture-release performance of the thylakoid components maintained good reproducibility after multiple cycles of treatment. The results in Fig. 3E demonstrated that the laser groups on the 3rd and 14th days of storage both exhibited a characteristic shift of the fluorescence peak, indicating that the fluorescence response spectral characteristics associated with ^1^O_2_ under near-infrared irradiation remained unchanged after storage of the thylakoids for different durations. Fig. 3F further showed that no significant difference was detected in the changes in fluorescence intensity at various time points within 0-14 days, confirming that the near-infrared-triggered ^1^O_2_ capture-release performance of thylakoid components did not attenuate during the 14-day storage period, thus exhibiting excellent long-term functional stability.

The ^1^O_2_ generation ability of CM-Thy under the laser irradiation was assessed by measuring the attenuation of the absorption value of the ^1^O_2_ probe DPBF at 415 nm [105,106]. The results are presented in Fig. 3, where Figure G illustrates the absorbance change curve of the mixture containing CM-Thy and DPBF. Within 10 min of irradiation, DPBF exhibited a high degradation rate which became more pronounced with increasing laser irradiation time. As indicated by the prominent characteristic absorption peak of chloroplasts (purple arrow), it can be inferred that CM-Thy possesses the capability to produce ^1^O_2_, and its production increases with longer irradiation periods. Fig. 5H displays negligible degradation in the absorbance change curve of DPBF under simple illumination using a 660 nm laser at different time intervals, suggesting that solely relying on this wavelength does not generate significant amounts of ^1^O_2_. Fig. 5I demonstrates how both DPBF alone and DPBF combined with CM-Thy exhibit changes in absorbance as a function of laser irradiation time at a wavelength of 415 nm. It is visually evident from this figure that there is a minimal decrease in absorbance value under simple laser irradiation. However, within just 3 min of laser exposure, the absorbance value for DPBF mixed with CM-Thy decreased from an initial reading of 1.17 (±0.003) to 0.71 (±0.013). This indicates that most of the DPBF present in the mixture reacted with generated ROS and underwent degradation due to their interaction with produced ^1^O_2_. These findings highlight that Thy within CM-Thy retains its potent capacity for generating singlet oxygen species, thereby enabling tumor scavenging through reactive oxygen species production.

### 3.4 CM has homologous targeting to parental tumor cells

**Fig. 4:**
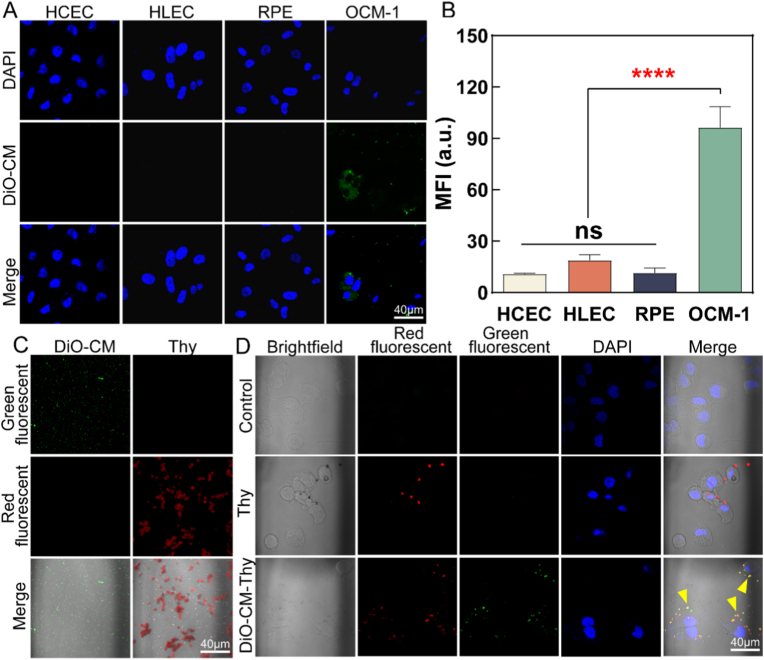
**Tumor targeting ability of CM-Thy and its uptake behavior by OCM-1 cells.** A: Homologous targeting analysis, representative fluorescence microscope images of DiO-CM and HCEC, HLEC, RPE, and OCM-1 cells. The blue signal represents the nucleus, and the green signal represents the DiO-labeled cell membrane. B: Quantitative statistics of green fluorescence signal in Figure A (n = 5). C: Pictures of DiO-CM and Thy in red and green fluorescent channels, where the merged image adds a bright field image. For contrast use Figure D. D: Representative fluorescence images of Thy and DiO-CM-Thy in OCM-1. Blue is the nucleus, green is DiO-CM, and red is Thy, Yellow arrow: CM-Thy. The magnification was 63 × for all images.

Further evidence supporting homologous targeting using OCM-derived cell vesicles was demonstrated on intraocular cells of HCECs, HLECs, RPEs, and OCM-1 due to their rich content of specific membrane proteins on cell surfaces and varying affinities towards different source membranes. As depicted in Fig. 4A, it is evident that OCM-1 cells exhibit enhanced uptake of membrane vesicles (DiO-CM) concurrently, with a predominant localization of the green fluorescence surrounding the nucleus (blue fluorescence signal). Fig. 4B represents a statistical analysis of the fluorescence intensity observed in Fig. 4A, revealing a significantly higher average fluorescence intensity for OCM-1 cells compared to the other three cell types (with statistically significant differences). These findings indicate that OCM-1-derived cell membrane vesicles (CM) demonstrate specific homotypic targeting behavior towards OCM-1 cells, underscoring their superior affinity for cognate cells and thereby enhancing the efficacy of modified drug delivery.

**Fig. 5:**
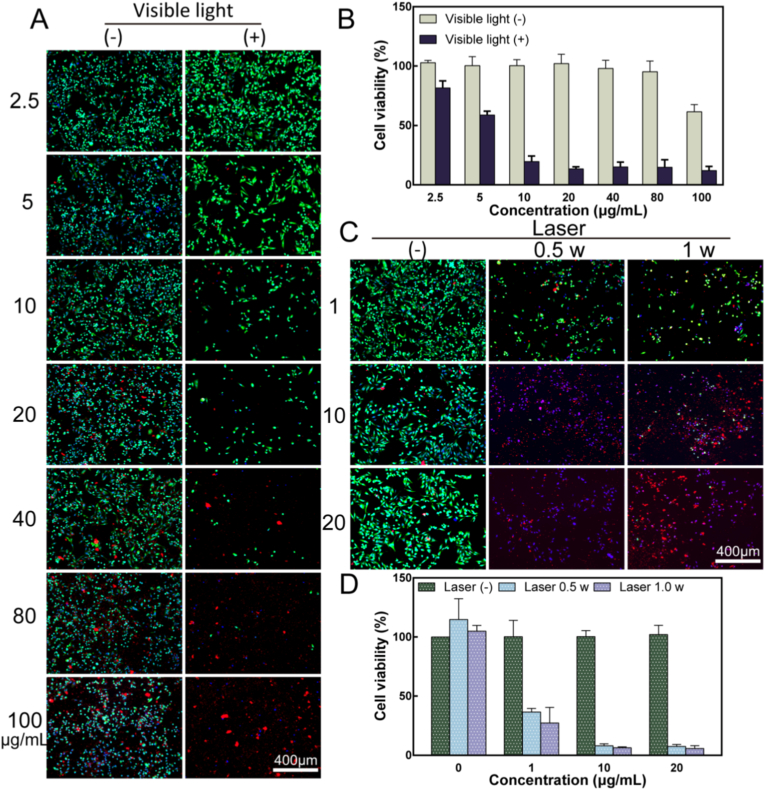
**The PDT effect of Thy.** A: Calcein AM/PI/Hoechst33342 staining of OCM-1 cells with different concentrations of Thy treated with light shielding and visible light. B: Statistical results of OCM-1 cell viability under different concentrations of Thy treated with light avoidance and visible light (n = 5). C: Calcein AM/PI/Hoechst33342 staining representation of OCM-1 cells after 3 min of different concentrations of Thy under light shielding and 660 nm laser irradiation, with laser power of 0.5 W/cm^2^ and 1 W/cm^2^. D: Statistical results of OCM-1 cell viability after 3 min irradiation with different concentrations of Thy and 660 nm laser (n = 5). The magnification was 10 × for all images.

### 3.5 CM effectively improves intracellular uptake

DiO-labeled CM was incubated with OCM-1 cells while nuclei were stained blue using DAPI. Additionally, separate observations were made on DiO-CM and Thy samples as controls. As illustrated in Fig. 4C, DiO-CM exhibits dispersed star-like green fluorescence throughout the entire field of view, whereas Thy displays multiple elliptical clusters due to film fusion. Fig. 4D demonstrates individual Thy adhering to the membrane surface of OCM-1. However, only a few instances are visible within the field of view and no adhesion around individual cells is observed. In the DiO-CM-Thy group, yellow fluorescence emerges from overlapping green fluorescence emitted by DiO-CM and red fluorescence emitted by Thy, and this yellow signal penetrates the cytoplasm of OCM-1 cells within the visual field. This outcome suggests the successful internalization of DiO-CM-Thy by homologous OCM-1 cells facilitating subsequent generation of ROS upon excitation by light sources. This homologous-targeting adhesion ability may stem from multiple membrane proteins, such as E-cadherin (which is mainly involved in intercellular adhesion) and CD81 (which participates in intercellular interactions) as shown in Fig. S5.

### 3.6 The daily light source can drive the anti-tumor behavior of Thy

Before evaluating the efficacy of CM-Thy in PDT for choroidal melanoma, this part first assessed the intrinsic cytotoxicity of Thy. During this process, it was discovered that daily light exposure could enhance the tumor-killing effect of Thy. In Fig. 5A, it is evident that even with increasing concentrations of Thy (indicated by spontaneous red fluorescence), there are still a considerable number of surviving cells (overlapping green and blue regions) under light avoidance conditions. This confirms that within the selected concentration range, Thy exhibits good biocompatibility. However, when exposed to visible light, the number of viable cells significantly decreased and some weaker cells lost their adhesion properties and were eliminated during staining steps. In higher concentration groups of Thy, a distinct smaller form of red fluorescence appeared alongside Thy fluorescence, further confirming that visible light can enhance the anti-tumor effects of Thy on OCM-1 cells. This discovery highlights daily light as a potential factor in disease treatment and may lead to milder therapeutic approaches.

Fig. 5B presents quantitative statistics on OCM-1 cell viability under two treatment methods. It can be observed that cell viability is better when using less than 100 μg/mL of Thy. However, cell viability decreases with increasing concentrations under visible light irradiation. At a concentration level as low as 5 μg/mL, cell viability drops below 80%, promoting apoptosis in tumor cells.

To shorten treatment time, PDT was performed using a laser at 660 nm wavelength on the cells. As shown in Fig. 5C, under dark conditions, the cellular state resembles Fig. 5A where cell density significantly decreases at concentrations above 1 μg/mL or more apoptotic cells appear at concentrations exceeding 10 μg/mL when subjected to either 0.5 W/cm^2^ or 1 W/cm^2^ irradiance levels respectively. The broken cells exhibit purple fluorescence (the red fluorescence of PI overlaps with the blue fluorescence of Hoechst33342). Quantitative analysis in Fig. 5D also confirmed that laser irradiation at both powers could drive Thy to kill OCM-1 cells. Notably, the study additionally investigated the photothermal phenomena of each component of CM-Thy. As shown in Fig. S7, under 660 nm laser irradiation at a power density of 0.5 W/cm^2^, no significant changes were observed in the thermal imaging of PBS, CM and Thy at different concentrations, with the temperature stabilizing at approximately 25 °C, indicating the absence of photothermal effects in all these groups. In contrast, under 1 W/cm^2^ laser irradiation, PBS and CM still exhibited no obvious response, whereas Thy showed a concentration-dependent photothermal effect, a slight temperature increase was detected in the 20 μg/mL group, while the temperature in the 40 μg/mL group rose significantly to nearly 40 °C, and the color of the thermal images gradually brightened over time [107]. Only at this point could the temperature approach the moderate hyperthermia range, thereby exerting an inhibitory effect on tumor cell growth. Therefore, the therapeutic concentration selected in subsequent experiments 10 μg/mL mainly relies on PDT rather than triggering the photothermal mechanism, which renders the therapeutic process safer and more comfortable.

### 3.7 Changes in various aspects of cell apoptosis induced by CM-Thy

#### 3.7.1 Intracellular ROS detection

**Fig. 6:**
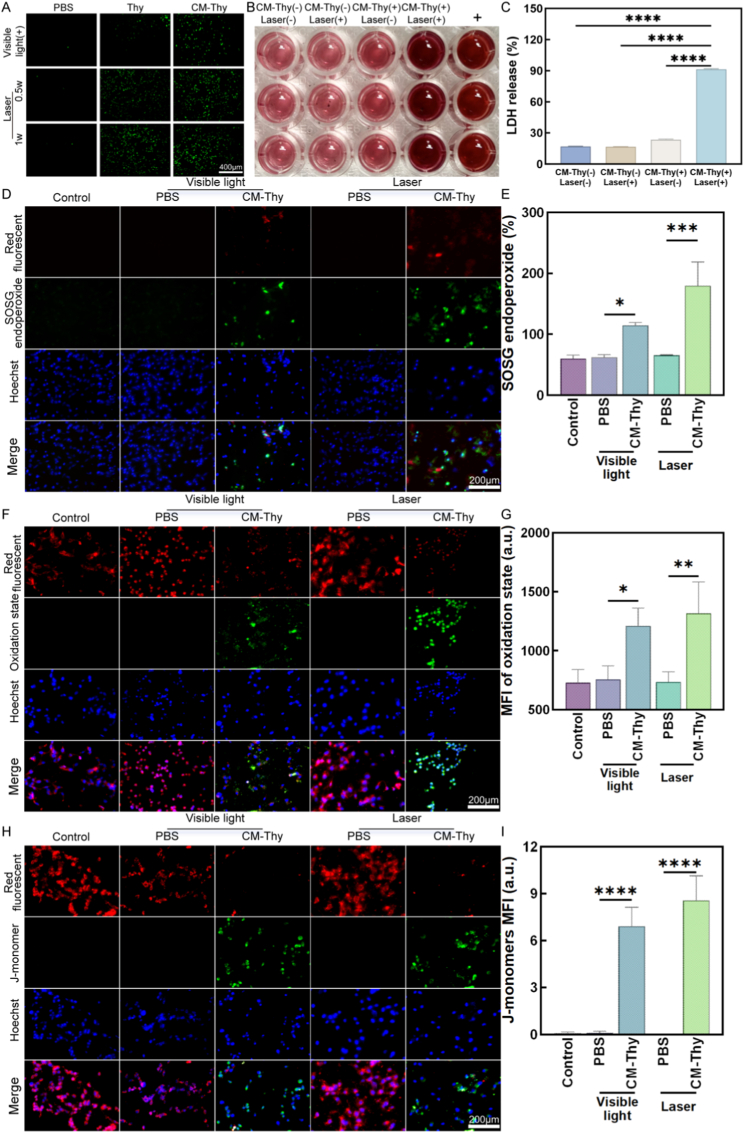
**Changes in various aspects of cell apoptosis induced by CM-Thy.** A: DCFH-DA staining images of ROS released by Thy and CM-Thy after light processing with different power. The green signal represents intracellular ROS marked by the probe. The magnification was 10 × for all images. B: Color response diagram of LDH release detection: control group (CM-Thy- Laser -), Laser group (CM-Thy- Laser+), CM-Thy group (CM-Thy + Laser-) and PDT group (CM-Thy + Laser+). C: Calculated statistics of LDH release rate corresponding to Figure B (n = 3). D: Intracellular singlet oxygen generation under visible light and laser irradiation (green fluorescence: SOSG; red fluorescence: CM-Thy; blue fluorescence: cell nucleus). E: Quantitative analysis of SOSG endoperoxide fluorescence intensity in Figure D (n = 5). F: Intracellular lipid peroxidation level under visible light and laser irradiation (green fluorescence: oxidized BDPY; red fluorescence: CM-Thy and reduced BDPY; blue fluorescence: cell nucleus). G: Quantitative analysis of oxidized BDPY fluorescence intensity in Figure F (n = 5). H: Changes in intracellular mitochondrial membrane potential under visible light and laser irradiation (green fluorescence: mitochondrial monomers; red fluorescence: CM-Thy and mitochondrial polymers; blue fluorescence: cell nucleus). I: Quantitative analysis of mitochondrial monomer fluorescence intensity in Figure H (n = 5). Images in Figures D, F and H were acquired under a 20 × objective.

The photodynamic properties of CM-Thy were demonstrated by detecting the generated ROS using a DCFH probe. As depicted in Fig. 6A, cells in the PBS/DCFH solution control group exhibited minimal spontaneous ROS production under all conditions. The ROS generation induced by Thy and CM-Thy with light exposure was significantly higher than that of the control group, indicating that CM enhanced the PDT effect of Thy under visible light. This enhancement could be attributed to the increased intracellular uptake of Thy facilitated by homologous targeting mediated by CM. However, at 660 nm laser irradiation, the ROS produced by the CM-Thy group was comparable to that of Thy due to the strong light acquisition ability of Thy in the near-infrared region (650-800 nm), which overshadowed the advantages conferred by CM.

#### 3.7.2 Damage to the cell membrane structure

ROS can induce cell death by disrupting the stability and integrity of cellular membranes. The integrity of the cell membrane is reflected by intracellular lactate dehydrogenase (LDH) levels [69]. Therefore, assessment of LDH release plays a crucial role in evaluating the extent of cell damage. By observing color development as shown in Fig. 6B, the control group (CM-Thy- Laser-), Laser group (CM-Thy- Laser+), and CM-Thy group (CM-Thy + Laser-) displayed a light pink appearance when reacting with reagents. After CM-Thy Laser irradiation (CM-Thy + Laser+), a dark red reaction appeared, which was deeper than the orange-red appearance observed in positive control groups. When setting absorbance from positive control as 100% (Fig. 6C), LDH release rates for these three groups were approximately 20%. Upon laser excitation, this release rate increased significantly. However, the damage to the cell membrane structure caused by secondary necrosis, pyroptosis, and other processes during cell apoptosis leads to the release of intracellular enzymes into the extracellular space.

#### 3.7.3 Generation of intracellular singlet oxygen

Fig. 6D and E collectively illustrate the results of ^1^O_2_ generation induced by CM-Thy under different light conditions. As shown in Fig. 6D, no detectable SOSG green fluorescence signal or CM-Thy red fluorescence signal was observed in the control group. Under visible light irradiation, no relevant signals were detected in the PBS group, while only a small amount of SOSG green fluorescence signal and the corresponding CM-Thy red fluorescence signal appeared in the CM-Thy group. Under laser irradiation, no signals were found in the PBS group, whereas the SOSG green fluorescence signal was significantly enhanced in the CM-Thy group. The quantitative analysis of the peroxide ratio in SOSG in Fig. 6E further verified that the SOSG ratio in the CM-Thy group was higher than those in the control and PBS groups under all light conditions. Therefore, CM-Thy can significantly promote ^1^O_2_ generation triggered by visible light and laser irradiation.

#### 3.7.4 Change in the degree of intracellular lipid peroxidation

In Fig. 6F, the green fluorescence corresponds to the detection signal of lipid peroxidation. No green fluorescence was observed in either the control group or the PBS group under light conditions, in contrast, distinct green fluorescence was detected in the CM-Thy group with light treatment, indicating that CM-Thy can induce cellular lipid peroxidation under light irradiation. The quantitative results in Fig. 6G further verified that the mean fluorescence intensity reflecting the lipid peroxidation status in the CM-Thy group under light conditions was significantly higher than those in the control group and the PBS group under light conditions, which confirmed the inductive effect of CM-Thy on cellular lipid peroxidation under light irradiation from a quantitative perspective. Combined with the aforementioned results, CM-Thy generates ^1^O_2_ upon light irradiation. As a highly reactive reactive oxygen species, ^1^O_2_ readily attacks the unsaturated fatty acid chains in the cell membrane, thereby triggering lipid peroxidation reactions [108,109], which constitutes the core mechanism underlying the appearance of green lipid peroxidation signals in the CM-Thy group under light conditions in this experiment.

#### 3.7.5 Change in cellular mitochondrial membrane potential

In the fluorescence imaging of Fig. 6H, the green fluorescence corresponds to mitochondrial monomers, which reflects decreased mitochondrial membrane potential (early cellular apoptosis) [110]. No obvious green fluorescence signal was observed in either the control group or the PBS group under light conditions; in contrast, distinct green fluorescence was detected in the CM-Thy group with light treatment, suggesting that the presence of CM-Thy under light irradiation induces a decrease in mitochondrial membrane potential in cells. The quantitative results in Fig. 6I further demonstrated that the mean fluorescence intensity of monomers in the CM-Thy group under light conditions was extremely significantly higher than those in the control group and the PBS group under light conditions, which confirmed the inductive effect of CM-Thy on early cellular apoptosis under light irradiation from a quantitative perspective. This phenomenon is attributed to the fact that singlet oxygen generated by CM-Thy upon light irradiation can further damage the structure and function of the mitochondrial membrane, leading to a decrease in mitochondrial membrane potential and inducing early cellular apoptosis.

### 3.8 Proliferation capacity and pyroptosis mechanism of tumor cells

**Fig. 7:**
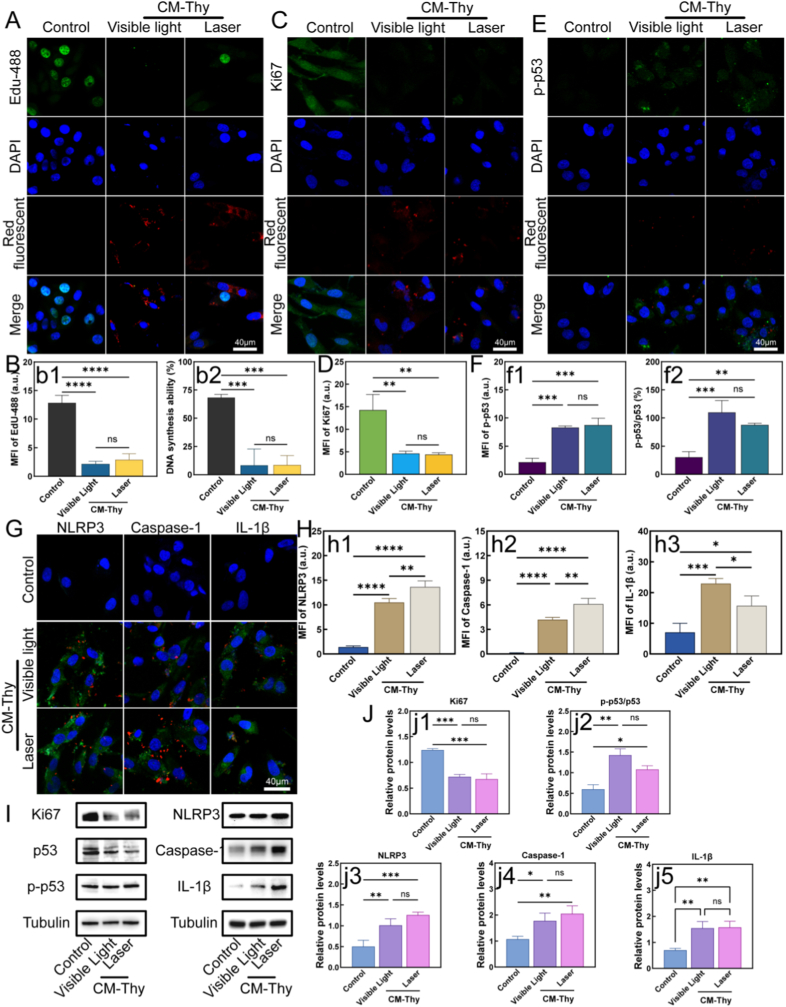
**Characterization of CM-Thy-mediated tumor cell proliferation inhibition, apoptosis activation, and NLRP3 inflammasome pathway activation under light irradiation.** A, B: EdU staining for detecting tumor cell DNA synthesis capacity, A: Fluorescence images (green: EdU-positive signals; blue: nuclear staining), B: Quantitative analysis of EdU mean fluorescence intensity (n = 3). C, D: Ki67 immunofluorescence staining for detecting cell proliferation activity; C: Fluorescence images (green: Ki67-positive signals; blue: nuclear staining); D: Quantitative analysis of Ki67 (n = 3). E, F: p-p53 immunofluorescence staining for detecting apoptotic pathway activation level, E: Fluorescence images (green: p-p53-positive signals; blue: nuclear staining), F: Quantitative analysis of p-p53 and p-p53/p53 (n = 3). G, H: NLRP3, Caspase-1, and IL-1β immunofluorescence staining for detecting the expression of inflammasome pathway-related proteins, G: Fluorescence images (green: target protein-positive signals; blue: nuclear staining), H: Quantitative analysis of each protein (n = 3).I, J: Western blot analysis for detecting the protein expression levels and semi-quantitative analysis of NLRP3, Caspase-1, and mature IL-1β (n = 3). The magnification was 63 × for all images.

Fig. 7 systematically investigated the killing effect of CM-Thy on the tumor cells after light irradiation. In terms of cell proliferation, EdU staining results in Fig. 7A and B demonstrated that cells in the control group and the light-only group exhibited dense green fluorescent signals, indicating active DNA synthesis and robust proliferation capacity [111]. In contrast, in the CM-Thy group treated with light irradiation, the EdU green fluorescent signals almost completely disappeared, and the DNA synthesis capacity showed an extremely significant decrease compared with the control group. Meanwhile, the Ki67 immunofluorescence results (a broad-spectrum proliferation marker) in Fig. 7C and D also revealed that the green fluorescent signal of Ki67 in the control group was strong, while the mean fluorescence intensity of this signal in the light-treated CM-Thy group was significantly reduced.

When DNA damage accumulates continuously, it phosphorylates downstream p53 protein and initiates the apoptotic program [112]. The detection results of the apoptosis-related protein p-p53 in Fig. 7E and F showed that the green fluorescent signal of p-p53 in the control group was very weak, whereas the signal was enhanced in the light-treated CM-Thy group. Meanwhile, the ratio of p-p53 to total p53 (p-p53/p53) was also significantly increased, directly demonstrating that p53-mediated programmed apoptosis was activated.

Excessive ^1^O_2_ generated by CM-Thy after the light irradiation can induce calcium overload and mitochondrial ROS accumulation, which activate the NLRP3 inflammasome in the cytoplasm to mediate pyroptosis [113,114]. The detection results of the NLRP3 inflammasome and its downstream effector molecules in Fig. 7G, H, 7I, and 7J showed that almost no green fluorescent signals of NLRP3, Caspase-1, and IL-1β were detected in the control group, while the fluorescent intensities of these three proteins were significantly increased in the light-treated CM-Thy group. Western blot protein quantification results further verified this trend, the protein expression levels of NLRP3, Caspase-1, and mature IL-1β were all significantly upregulated in the light-treated CM-Thy group. These findings fully indicated that the NLRP3 inflammasome pathway was strongly activated, thereby triggering inflammation-related pyroptosis and cell damage.

Collectively, the core mechanism by which CM-Thy kills tumor cells after light irradiation, as revealed in Fig. 7, is as follows: as a photosensitizer, CM-Thy can generate a large amount of highly ^1^O_2_ under light conditions. This ^1^O_2_ strongly inhibits tumor cell proliferation by directly interfering with cellular DNA replication and cycle progression. Simultaneously, it activates the classical p53 apoptotic pathway, initiates the programmed cell death program, and triggers NLRP3 inflammasome-mediated pyroptosis to further amplify cell damage. Eventually, a synergistic killing network involving oxidative stress, proliferation inhibition, and pyroptosis is formed.

### 3.9 The study on the inhibition mechanism of tumor formation of CM-Thy

As a solid tumor, the nutrient supply of choroidal melanoma tissues relies on tumor microcirculation, which includes existing blood vessels, neovascularization (NV), and vasculogenic mimicry (VM) [115]. The formation of NV primarily involves the sprouting of existing endothelial cells to generate new tumor capillaries, which entails the regeneration of endothelial cells from bone marrow-derived endothelial progenitors. Vascular density within the tumor is locally induced and unevenly distributed throughout the tumor, and it has been demonstrated that vascular density is associated with metastatic death in choroidal melanoma [116]. VM refers to an extracellular matrix-directed circulation characterized by vaso-like channels formed through cellular deformation, increased migration ability, and remodeling of the extracellular matrix. It differs from classical angiogenesis. VM has been shown to be associated with aggressive tumor growth patterns, as these channels provide a diffuse surface for the continued growth of choroidal melanoma [117]. This part aims to investigate whether treatment with CM-Thy can inhibit the mechanisms underlying choroidal melanoma formation.

#### 3.9.1 Study on the inhibition of hypoxia-induced VM phenomenon

**Fig. 8:**
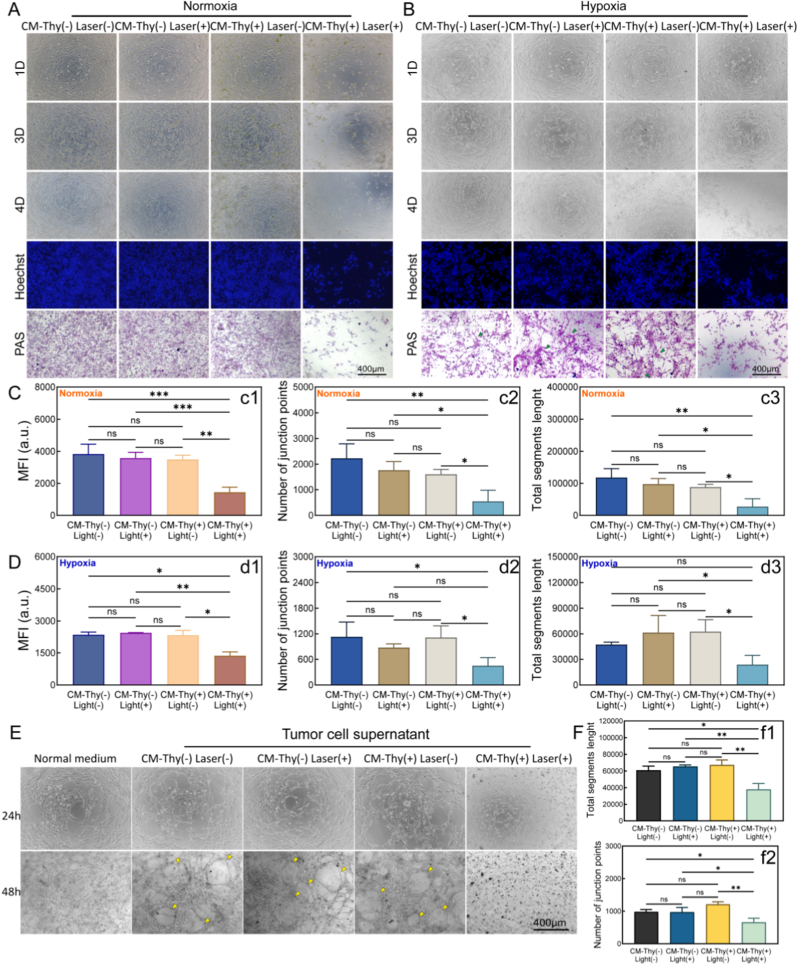
**Regulatory Effects of CM-Thy Combined with Light Irradiation on Vasculogenic Mimicry and Angiogenesis Under Different Oxygen Environments.** A and B: Vasculogenic mimicry of OCM-1 cells induced by different oxygen concentrations. In addition to continuous bright field observation, the cells were also stained with Hoechst33342 and PAS. Green arrow: positive structure, Magnification: 10 × . C: Quantitative analysis of vasculogenic mimicry under normoxic conditions (n = 3); c1: quantification of mean fluorescence intensity (MFI); c2: statistics of junction number; c3: statistics of total segment length. D: Quantitative analysis of vasculogenic mimicry under hypoxic conditions (n = 3). E: Establishment of HUVEC angiogenesis model and treatment results of different treatment groups. Yellow arrow: vessel-like structure. F: Quantitative analysis of angiogenesis (n = 3); f1: statistics of total vessel length; f2: statistics of vessel junction number. Magnification: 10 × .

Fig. 8A illustrates that under normal oxygen concentration conditions, there was an increase in tumor cell density in both the control group and laser group compared to the Thy group where normal growth was observed. Fig. 8B demonstrates that under hypoxic conditions, channel-like blank areas appeared in the arrangement of tumor cells. To obtain a clearer view of cell arrangement, nuclei were fluorescently labeled. Blue fluorescence covered the entire field of view under normal oxygen conditions while channel-like connections were observed under low oxygen conditions. In PAS staining, cells cultured with low oxygen showed more positive cytoplasm (green triangle mark), while cells cultured with conventional oxygen showed lighter cytoplasm. The tumor cells mimic the body's angiogenesis under low oxygen conditions and form tumor cell lines and tubes. Fig. 8C and D further confirmed the aforementioned hypothesis by analyzing angiogenesis-related indicators (junctions and segment lengths). For the CM-Thy group, no matter what concentration of oxygen culture, the number of tumor cells decreased, and the cell growth showed a flaky distribution. This phenomenon confirms that there may be other inhibitory mechanisms besides reducing the growth of VM.

#### 3.9.2 Study on the inhibition of endothelial cell angiogenesis

The experimental results of endothelial cell angiogenesis are presented in Fig. 8E. HUVEC cells were initially cultured in conventional media for 24 h, followed by complete coverage of the visual field after 48 h. After referring to the quantitative analysis (Fig. 8F), in the control group, laser group, and CM-Thy group, the OCM-1 cell culture supernatant significantly induced reticular vascular structure formation in HUVEC cells. At 24 h, HUVEC cells exhibited a network structure that became more pronounced at 48 h (indicated by yellow arrows). This phenomenon is attributed to cytokines secreted by tumor cells during growth that promote blood vessel development. However, tumor supernatants derived from the PDT group did not induce tubularization of HUVEC possibly due to a lack of relevant components necessary for endothelial cell growth and migration provided by treated tumor cells. This confirms that PDT therapy with CM-Thy effectively interferes with angiogenesis in HUVEC cells.

#### 3.9.3 Study on the inhibition of the migration behavior of tumor cells

**Fig. 9:**
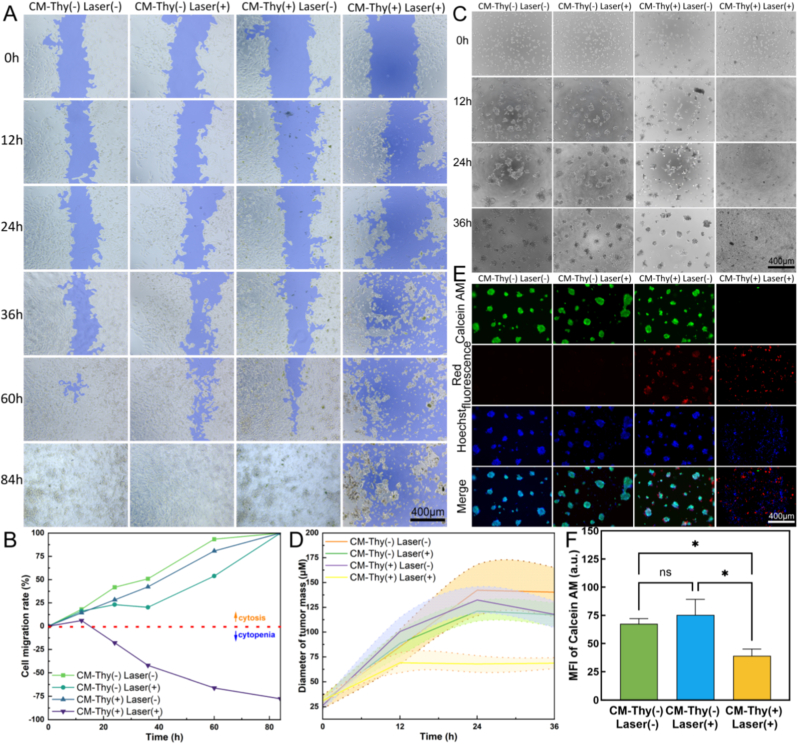
**Analysis of OCM-1 cell migration and tumor formation.** A: Continuous observation of OCM-1 cell migration process in different treatment groups. B: Statistical analysis of cell mobility in each group in the scratch test. C: The bright field images of OCM-1 in 3D tumor formation experiments of different treatment groups. D: Diameters of tumor masses in each group in Figure C (n = 3). E: Calcein AM/PI/Hoechst33342 staining of OCM-1 in 3D tumor formation experiments of different treatment groups. F: Quantitative statistical results of green fluorescence in tumor cells of each group in Figure E (n = 3). Magnification: 10 × .

The control group (CM-Thy- Laser-), Laser group (CM-Thy- Laser+), and CM-Thy group (CM-Thy + Laser-) demonstrated complete coverage of the scratched area within 84 h, as depicted in Fig. 9A. However, in the PDT group (CM-Thy + Laser+), the scratch area initially decreased and then gradually increased at 12 h, accompanied by a fragmented distribution of cells. In terms of cell migration efficiency shown in Fig. 9B, all groups except for the PDT group exhibited a gradual increase in cell mobility until reaching 100%. Conversely, the PDT group displayed an initial increase in migration above the red dashed line followed by a negative value below it, indicating a decrease in cell numbers. These findings suggest that CM-Thy can effectively inhibit cell migration upon excitation.

#### 3.9.4 Study on the inhibition to 3D tumor formation behavior

In Fig. 9C, OCM-1 cells progressively migrated to form tumor cell masses on the surface of the matrix gel with notable prominence at 36 h of culture (the central shadow represents cellular superposition). However, when subjected to PDT treatment, OCM-1 cells exhibited diffuse distribution without any tendency toward clumping. The diameter of tumor masses in each group was statistically analyzed, Fig. 9D demonstrates that tumor growth was significantly inhibited after laser treatment. Fig. 9E using Calcein AM/PI/Hoechst revealed stronger green fluorescence and cluster growth indicative of good cellular condition in the PBS group, whereas almost no surviving cells were observed (green fluorescence) along with fragmented blue fluorescence in the PDT group (CM-Thy Laser). Quantitative analysis of Fig. 9F also confirmed that tumor cell viability was significantly decreased. This confirms that CM-Thy-based PDT therapy effectively inhibits cellular clumping tendencies.

### 3.10 Establishment of choroidal melanoma model and results of intraocular treatment

**Fig. 10:**
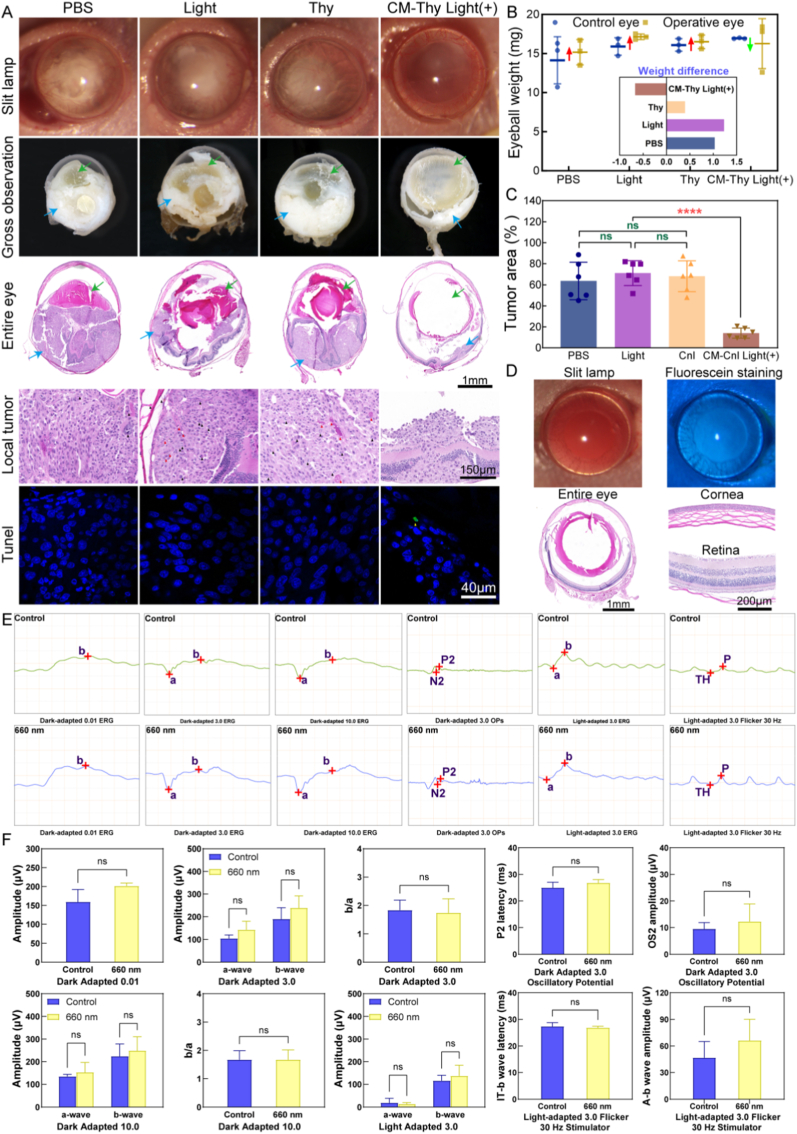
**In vivo evaluation of antitumor efficacy and biosafety.** A: Slit-lamp images, sagittal profile anatomical images, HE staining of the whole eye, and local tumor tissue and TUNEL staining results of nude mice in each group after treatment. Green arrow: lens; Blue arrow: tumor tissue. B: Weight and difference statistics of the eyes of nude mice in each group after treatment (n = 3). C:The ratio of the tumor area to the vitreous cavity in the eye (n = 6). D: Safety observation results of 660 nm laser treatment on mouse eyeballs, including slit-lamp images, fluorescein sodium staining images, and HE staining images. E, F: Changes in retinal electrophysiology and statistical analysis of characteristic value variations under various modalities after 660 nm laser irradiation (n = 3).

The results of each experimental group are presented in Fig. 10A. Slit-lamp examination revealed the presence of conspicuous white masses in the PBS group. Meantime, in the gross observation and HE images, the tumor tissue grew from the subretinal cavity and eventually filled the entire vitreous cavity while adhering to the lens surface. Similar observations were made in the light group and Thy group, resembling those of the modeling group. To conduct quantitative analysis of tumor growth, we statistically analyzed the tumor area and ocular volume in the eye section images of each experimental group.

Fig. 10B illustrates the weight statistics of separated eyeballs. The weight of the operated eye (right eye) slightly increased compared to the control eye (left eye) in the PBS-treated tumor model group, the Light-only group, and the Thy-only group. However, there was a slight decrease in the weight observed in the PDT (CM-Thy Light+) group's operated eye. When comparing eyeball weights between surgical eyes and control eyes, all groups showed positive differences except for the negative difference observed in the PDT treatment group's eyeball weight. This indicates that PDT has a certain therapeutic effect on a macro level.

As shown in Fig. 10C, there is no significant difference in the tumor area ratio among the PBS, Light, and Thy groups. However, there is a significant difference between these three groups and the treatment group (CM-Thy Light+). This result fully confirms that the treatment with CM-Thy can significantly inhibit the growth trend of tumors to a large extent. Local magnification images of tumors from all three groups exhibited characteristic mitotic phases (indicated by black arrows) and some red extracellular stromal-like structures (indicated by red arrows), indicating active proliferation and division of most tumor cells. However, in the PDT treatment group, only cloud-like tissues were observed under slit-lamp examination, accompanied by a significant reduction in tumor size upon sectional observation. Local observation revealed that tumors were predominantly confined to the subretinal cavity without the nuclear division phase present. TUNEL staining results showed green fluorescence (marked by yellow arrows) in the PDT treatment group, suggesting that this therapy promoted apoptosis of tumor cells. In addition, local tumor metastases were also found in the liver and lungs, which are prone to hematogenous metastasis in the animals (as indicated by the blue arrows in Fig. S8). However, this situation was not found in the treatment group (CM-Thy Light+). Overall, these findings confirm that CM-Thy PDT therapy exhibits potent anti-tumor effects and effectively reduces choroidal melanoma tissue volume.

In addition, the safety of the therapeutic modality was investigated. First, the cytotoxicity of CM-Thy and the laser (different powers) was tested on three intraocular cell samples, and both of these factors showed better cell safety in vitro (Fig. S9). The in vivo safety was subsequently explored. Fig. 10D demonstrates the safety assessment of 660 nm illumination. The slit-lamp image reveals a clear and transparent dioptric medium within the eye. Fluorescein sodium staining confirmed the absence of defects or obvious irritation on the ocular surface. HE staining analysis revealed no apparent abnormalities across all tissues within the whole eyeball specimen while maintaining distinct stratification between the cornea and retina without obvious damage. Fig. 10E and F presents the retinal electrophysiology results of the control group and the 660 nm light irradiation group, in Fig. 10E, the core waveforms (a-wave, b-wave) and oscillatory potential (op) subwaves of the two groups under dark and light adaptation conditions were consistent in morphology with no peak shift. Quantitative statistical analysis in Fig. 10F showed that there were no significant statistical differences in the core parameters such as amplitude, b/a ratio and latency between the two groups during the dark and light adaptation stages. In conclusion, 660 nm light irradiation did not exert a significant effect on the electrophysiological function of the retina, nor did it interfere with its signal transduction and functional activity. Therefore, the 660 nm laser irradiation parameters used in this study are safe, with no significant damage observed in retinal electrophysiological functions or ocular tissues, which is in line with the findings of previous related studies [118,119].

Furthermore, a hemolysis experiment was carried out to assess the hemocompatibility of CM-Thy. As illustrated in Fig. S10A, the solution of the positive control group (PC) appeared red, while distinct red blood cell sedimentation formed at the tube bottom in the Control, CM, Thy and CM-Thy groups, accompanied by transparent and colorless supernatants, which implied the lack of significant hemolytic activity. Quantitative determination of the hemolysis rate revealed that, as shown in Fig. S10B, the hemolysis rates in all the aforementioned groups were nearly 0%, confirming the non-hemolytic property of CM-Thy and verifying that this agent does not interfere with the physiological microenvironment of red blood cells during blood contact.

## 4 Conclusion

In recent years, the parental cell membrane has been utilized as a drug delivery carrier for tumor treatment based on the concept of "derived from cancer, used for cancer". In this study, we developed a naturally derived tumor cell membrane-chloroplast membrane system (CM-Thy) with inherent tumor-targeting capability and photodynamic activity. This therapeutic approach demonstrates excellent biological safety, and in vitro, experiments have confirmed the selective targeting property of CM-Thy towards OCM-1 tumor cells. Moreover, light-induced ROS production by Thy within tumor cells enhances the efficacy of photodynamic therapy. Singlet oxygen produced by CM-Thy after the light irradiation exerts dual effects, it not only directly causes oxidative damage to dna, but also disrupts the structural integrity of mitochondrial and cell membranes. Based on these damages, the generated oxidative damage signals further initiate pyroptosis via the NLRP3 inflammasome pathway, while simultaneously inducing programmed apoptosis of cells, ultimately exerting efficient cytotoxicity against target cells. However, it is worth noting that CM-Thy exerts inhibitory effects on mechanisms such as tumor angiogenesis, vasculogenic mimicry, 3D tumor formation, and migration of tumor cells. Furthermore, animal models of choroidal melanoma have shown a significant reduction in tumor volume following treatment with CM-Thy. Importantly, in eyes exposed to natural daily light conditions, intraocular CM-Thy can also be activated to facilitate PDT treatment. This study introduces a novel concept of inter-species biological therapy and holds promise for providing targeted and mild treatment options for intraocular tumors.

## CRediT authorship contribution statement

**Jiahao Wang:** Writing – original draft, Methodology, Investigation, Formal analysis, Data curation. **Zhirong Chen:** Validation, Investigation, Data curation. **Yuexin Yang:** Validation, Investigation. **Yajia Wang:** Writing – review & editing, Investigation. **Hongying Xie:** Writing – review & editing, Investigation. **Wenxin Hong:** Investigation. **Quankui Lin:** Writing – review & editing, Supervision, Resources, Project administration, Funding acquisition, Conceptualization.

## Ethics approval and consent to participate

The animal experiments were approved by the Committee and Laboratory Animal Center of Eye Hospital, Wenzhou Medical University. The ethical approval Number was YSG24061404.

## Declaration of competing interest

The authors have declared that no competing interest exists.

## Data Availability

Data will be made available on request.
