# Supplementary material for: Cell membrane-encased thylakoid as white light triggered PDT therapy for facile and targeted choroidal melanoma treatment
Source: Bioact Mater. 2026 Apr 1;63:31–55. doi: 10.1016/j.bioactmat.2026.03.058 (PMC13084682; doi:10.1016/j.bioactmat.2026.03.058)
Supplement: Multimedia component 1 [file mmc1.docx]

Supplementary Information

**Cell membrane-encased thylakoid as white light triggered PDT therapy for facile and targeted choroidal melanoma treatment**

**Experimental Section**

**Materials**

Phosphate-buffered saline (PBS) was purchased from Boster Biological Technology. The chloroplasts were isolated from fresh spinach purchased at the local supermarket using the method of an improved method like section 2.3. The OCM-1 cell membrane (CM) was extracted using the method of extrusion in section 2.4. The cell counting kit-8 (CCK-8) , Anti-fade Mounting Medium with DAPI, Mito-Tracker Red CMXRos, Lyso-Tracker Red and Calcein/PI Cell Viability/Cytotoxicity Assay Kit were purchased from Shanghai Biyuntian Biological Co. Dulbecco’s modified Eagle’s medium (DMEM), DMEM/F12 (1:1), DMEM/F-12 containing L-glutamine, Fetal bovine serum (FBS), 0.05% trypsin-EDTA, penicillin-streptomycin solution, and other cell culture-related reagents were purchased from Gibco. The antibodies of E-Cadherin, ZO-1, EpCAM, CD81, FN, and α-SMA for immunofluorescence were purchased from Thermo Fisher Scientific and Abcm. All the secondary antibodies were from Santa Cruz.

Human lens epithelial cells (HLECs) and retinal pigment epithelium cells (RPEs) were propagated in the DMEM/F-12-based complete medium. Human corneal epithelial cells (HCECs) were propagated in a DMEM/F-12-based complete medium containing L-glutamine. Every complete culture medium was supplemented with 10% FBS and 1% penicillin-streptomycin, and the cells were incubated at 37 ℃ with a CO_2_ concentration of 5%. The medium was changed every 2 to 3 days.

**Measurements**

**Identification of leaf extracts**

The prepared leaf extracts were diluted, and the absorption values at each wavelength were measured using an ultraviolet spectrophotometer (UV-1780, Shimadzu, Japan). Subsequently, the droplets were added to a slide and sealed, allowing for observation of their natural morphology under a phase contrast microscope (Axio Observer3, ZEISS, Germany). The prepared slides of leaf extracts were then subjected to fluorescence microscopy (DMi8, Leica, Germany) in order to observe the fluorescence effect.

The membrane protein properties of the extracts were qualitatively analyzed using the SDS-PAGE Coomassie bright blue staining method. In simple terms, after quantification of the BCA protein extract, it was mixed thoroughly with 5 × sample buffer according to a specific ratio and heated at 100 ℃ for 10 minutes to denature the protein. The protein sample and marker were sequentially added into the sample wells, followed by the application of voltage until the completion of electrophoresis. After electrophoresis, the gel was gently washed with deionized water before pouring off excess liquid. Approximately 20 mL of Coomassie Blue Fast Staining Solution was added onto the gel surface for staining purposes. Following staining, decolorization was carried out by adding deionized water in a room temperature shaker for 2 hours (with water changed every 10 minutes). Overnight decolorization with sufficient deionized water allowed for clearer visualization of protein bands on the gel surface which could be photographed and analyzed.

**Morphological Characterization of CM-Thy**

The surface morphology and three - dimensional structure of the CM-Thy hybrid membrane were observed using a transmission electron microscope (TEM, FEI Talos F200, USA).

**SDS-PAGE Coomassie bright blue staining**

Proteins from OCM-1 cell protein along with CM-Thy and Thy were analyzed by SDS-PAGE Coomassie bright blue staining. After quantification of the BCA protein extract, it was mixed thoroughly with 5×sample buffer according to a specific ratio and heated at 100 ℃ for 10 minutes to denature the protein. The protein sample and marker were sequentially added into the sample wells, followed by the application of voltage until the completion of electrophoresis. After electrophoresis, the gel was gently washed with deionized water before pouring off excess liquid. Approximately 20 mL of Coomassie Blue Fast Staining Solution was added onto the gel surface for staining purposes. Following staining, decolorization was carried out by adding deionized water in a room temperature shaker for 2 hours (with water changed every 10 minutes). Overnight decolorization with sufficient deionized water allowed for clearer visualization of protein bands on the gel surface which could be photographed and analyzed.

**Membrane Protein and Organelle Analysis**

OCM-1 cells were cultured on cell slides with a diameter of 14 mm. After a day's incubation, the cells were fixed with 4% pre-chilled paraformaldehyde for 30 minutes. The extracted cell membranes (CM) were subjected to centrifugation at 12,000 rpm for 10 minutes and then resuspended in paraformaldehyde for fixation in the same manner. Following the fixation of both the cells and CM, they were rinsed thoroughly with PBS. Next, a blocking step was carried out using 5% BSA to minimize non-specific binding. Subsequently, the samples were incubated overnight at 4 ℃ with primary antibodies targeting E-Cadherin, ZO-1, EpCAM, CD81, FN, and α-SMA. Post-incubation, the samples were washed three times with PBS to remove unbound primary antibodies. Then, they were incubated with the appropriate secondary antibodies at room temperature for 1.5 hours. Finally, an anti-fade mounting medium containing DAPI was added, and coverslips were placed on top to complete the sample preparation. Images of each fluorescence channel were then captured using a confocal laser scanning microscope (Zeiss LSM 880, Germany).

To ascertain the purity of the extracted cell membranes, mitochondrial and lysosomal dyes were utilized to label DiO-CM. Firstly, the staining working solutions for mitochondria and lysosomes were freshly prepared. After DiO-CM was washed with PBS and centrifuged, Mito-Tracker Red CMXRos staining solution and Lyso-Tracker Red staining solution were added separately. The samples were then incubated in a 37 ℃ for 30 minutes. Post-incubation, the samples were washed twice with PBS to eliminate excess dye. Subsequently, the samples were resuspended in an anti-fade mounting medium containing DAPI. Finally, the samples were carefully dropped onto glass slides, covered with coverslips, and stored at -20 ℃ until they were ready for examination under a confocal microscope.

**Characterization of Photothermal Effect**

PBS, CM solution, and Thy solutions at concentrations of 20 μg/mL and 40 μg/mL were prepared separately. Equal volumes of each solution were transferred into a 96-well plate with triplicate wells set up for each group. Subsequently, the adjustable-power 660 nm laser irradiation device and infrared thermal imager were adjusted and calibrated. The samples were first irradiated with a laser at a power density of 0.5 W/cm^2^. Thermal images were acquired and temperature data were recorded at 0, 60, 120, and 180 s, respectively. Then, the samples were replaced with new ones from the same batch, the laser power density was adjusted to 1 W/cm^2^, and the aforementioned operations of thermal imaging acquisition and temperature recording at the same time points were repeated. Finally, thermal images at all time points were compiled, and temperature-time curves were plotted based on the recorded temperature data.

**Evaluation of tumor metastasis.**

After the animals were euthanized, organ samples such as the heart, liver, lung, kidney and spleen were collected from the nude mice in each experimental group. The operation should be rapid and the tissue integrity should be maintained. Subsequently, each tissue was fixed, dehydrated with gradient alcohol, transparent with xylene, impregnated with paraffin wax and embedded into wax blocks. Then the blocks were fixed on a microtome and cut into 8 - 10 μm thin sections. After staining with H&E, the sections were sealed with neutral resin. After the neutral resin dried, pictures were taken with a 3D slide scanner for observation to evaluate the metastasis of tumor tissues in each organ.

**Exploration of cytotoxicity in vitro**

In this study, the safety of CM-Thy and its effect on cells was investigated in vitro. HCECs, HLECs, and RPEs were cultured using the aforementioned cell culture methods and seeded into 96-well plates at a density of 5×10^3^/well (n=5). Each well was supplemented with 100 μL of complete culture medium and incubated overnight until cell attachment occurred. Subsequently, each group received either CM-Thy (at a concentration of 20 µg/mL) or an equivalent volume of PBS. The light group underwent irradiation for 3 minutes using laser irradiation powers of 0.5 W/cm^2^ and 1W/cm^2^ respectively. After irradiation, the cells were incubated with either the control group (PBS) or the CM-Thy group for 24 hours, followed by an evaluation of various factors' effects on different cell types using Calcein/PI staining. In simple terms, Calcein-AM and PI double fluorescence staining were employed to detect living and dead cell distribution in the plates; five parallel samples were set up in each group. During staining, working reagents (Calcein-AM reagent, PI reagent, and detection buffer mixed at a ratio of 1:1:1000) were added to each well in the 96-well plate at a volume of 100 μL/well. Subsequently incubated under light protection at a temperature of 37 ℃ for 30 minutes in a cell incubator. After staining, the samples were observed under a fluorescence microscope (DMi8, Leica, Germany) and photographed.

Additionally, CCK-8 reagents were used to quantitatively determine the viability of the three types of cells. The seeding procedure for each cell type remained unchanged, and five parallel samples were set up in each group. After 24 hours of culture, the existing media in the plates were sucked out and discarded. Then, the CCK-8 solution was prepared by mixing it with fresh media at a ratio of 1:10. Finally, this mixture was added to the 96-well cell culture plate with a volume of 100 μL per well. Another control group (fresh media mixed with CCK-8 solution) was set up to obtain the absorbance value of the blank holes. Under the condition of full protection from light, the cell culture plate with the dye solution was incubated in a cell incubator at 37 ℃ for 2 hours, and the absorbance value of each hole at 450 nm was detected with a multi-functional ultraviolet enzyme labeling instrument (SpectraMax 190, USA), and the data were recorded and processed and analyzed.

**Blood compatibility**

Initially, 8 ml of fresh rabbit blood was harvested in an anticoagulant tube and subsequently diluted with 10 mL of sterile physiological saline. The experimental design consisted of four groups, namely the negative control group (NC), positive control group (PC), Thy treatment group and CM-Thy treatment group. For sample preparation, the Control group was supplemented with physiological saline, the PC group was treated with equal volumes of distilled water, while the thy and CM-Thy groups were administered equal volumes of the respective material working solutions. All tubes were placed in a constant-temperature water bath at 37 ℃ for 60 min of pre-incubation. Following the slow and uniform addition of diluted fresh rabbit blood, the incubation was continued for an additional 2 h under the same conditions. Thereafter, the samples were subjected to centrifugation at a speed of 2000 rpm for 10 min, and the absorbance values of the resulting supernatants were detected at a wavelength of 545 nm using a microplate reader. Finally, the hemolysis rate of each group was calculated based on the measured absorbance data.

**
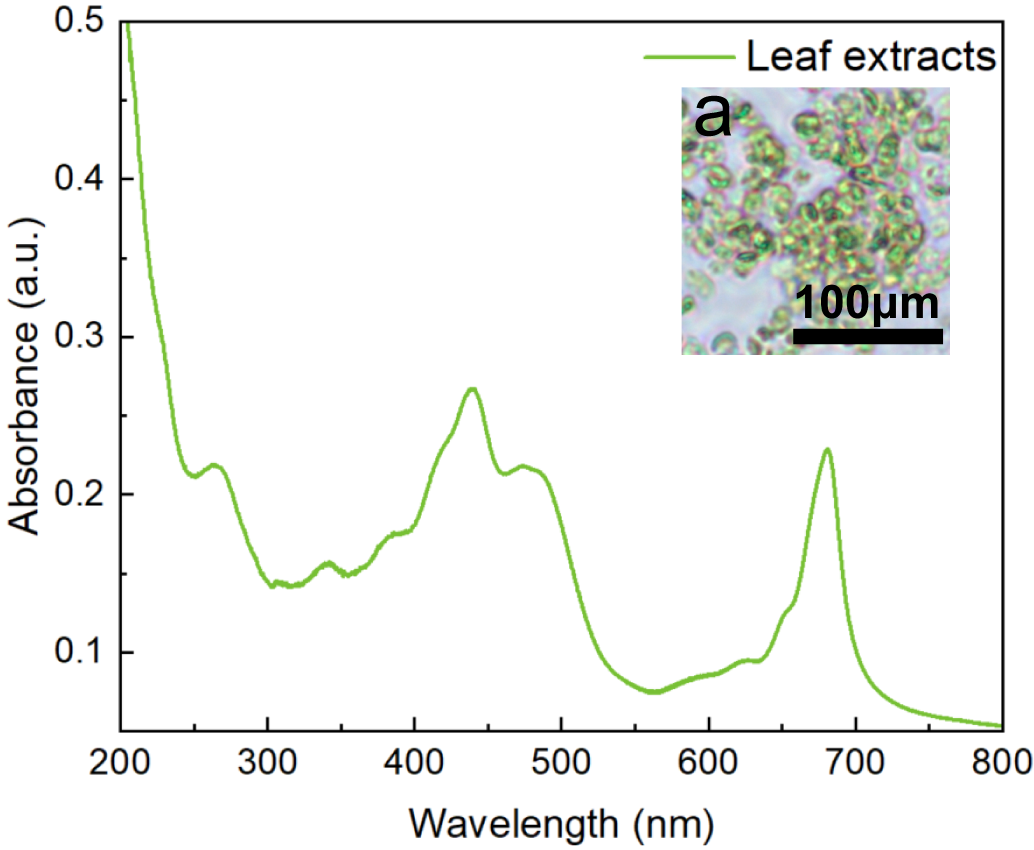
**

Figure S1 Ultraviolet absorption image of leaf extracts. Figure a shows the morphology of extracts in the brightfield.


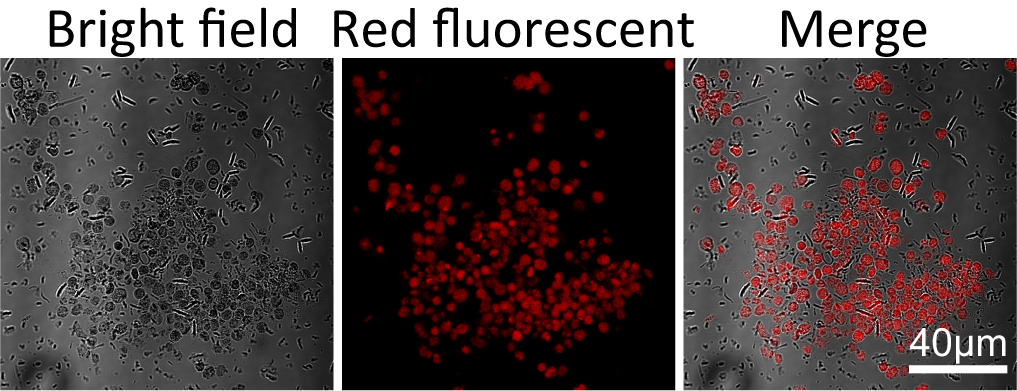


Figure S2 leaf extracts image under red fluorescence excitation. Magnification: 63×.


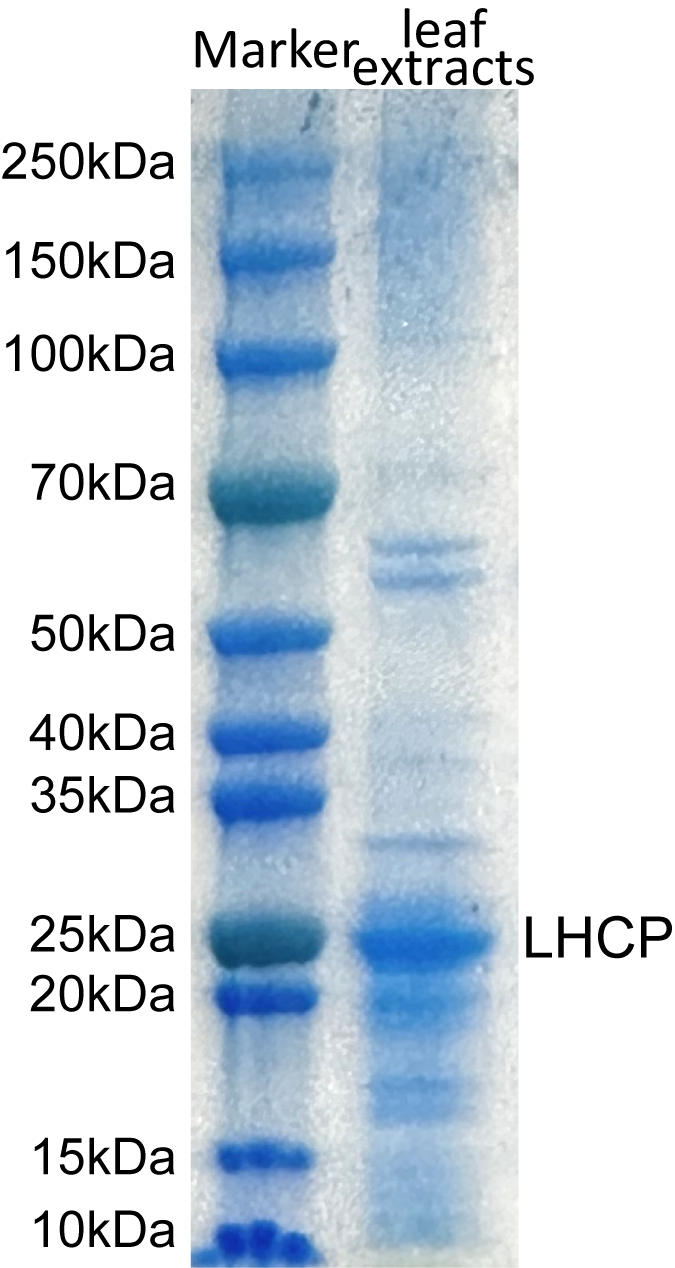


Figure S3 SDS-PAGE qualitative image of the protein of leaf extracts stained with Coomassie bright blue.


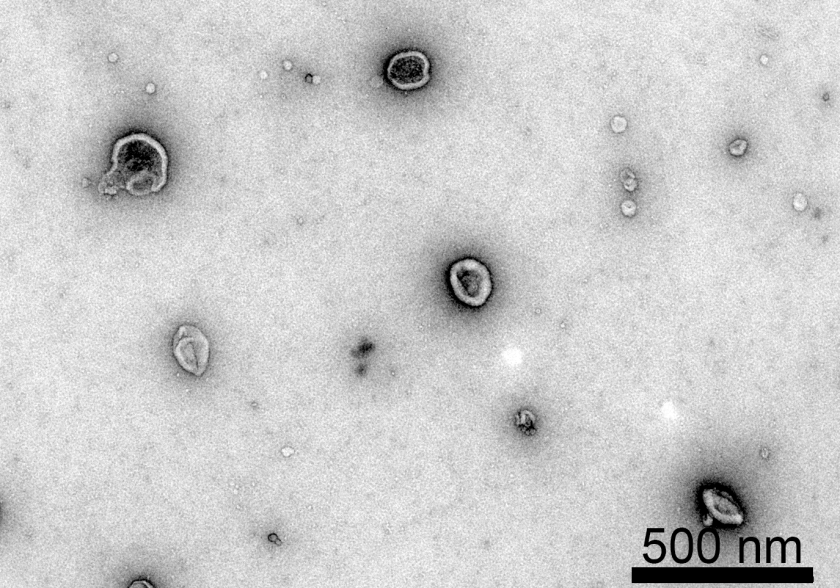


Figure S4 The large-scale TEM image of CM-Thy.

**
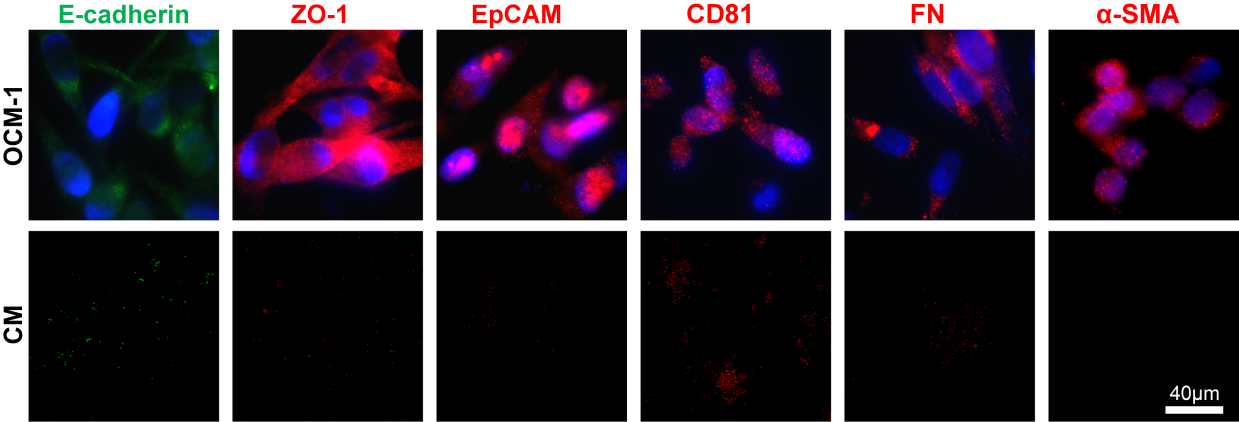
**

Figure S5 The expression status of the surface membrane proteins (E-Cadherin, ZO-1, EpCAM, CD81, FN) and the cytoskeletal protein (α-SMA) of OCM-1 cells and CM was investigated. Among them, E-Cadherin was presented in green, while the other proteins were shown in red. Magnification: 63×.


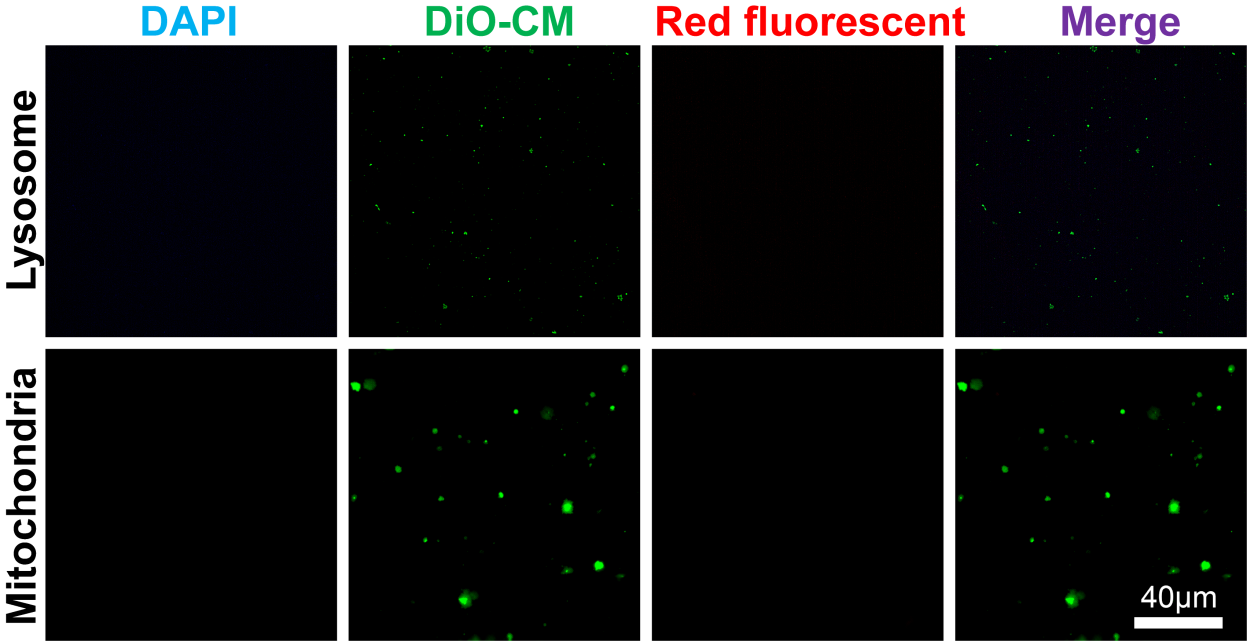


Figure S6 The expression status of lysosomes and mitochondria on DiO-CM was examined. The two types of organelles were labeled in red, DiO was shown in green, and DAPI was presented in blue. Magnification: 63×.


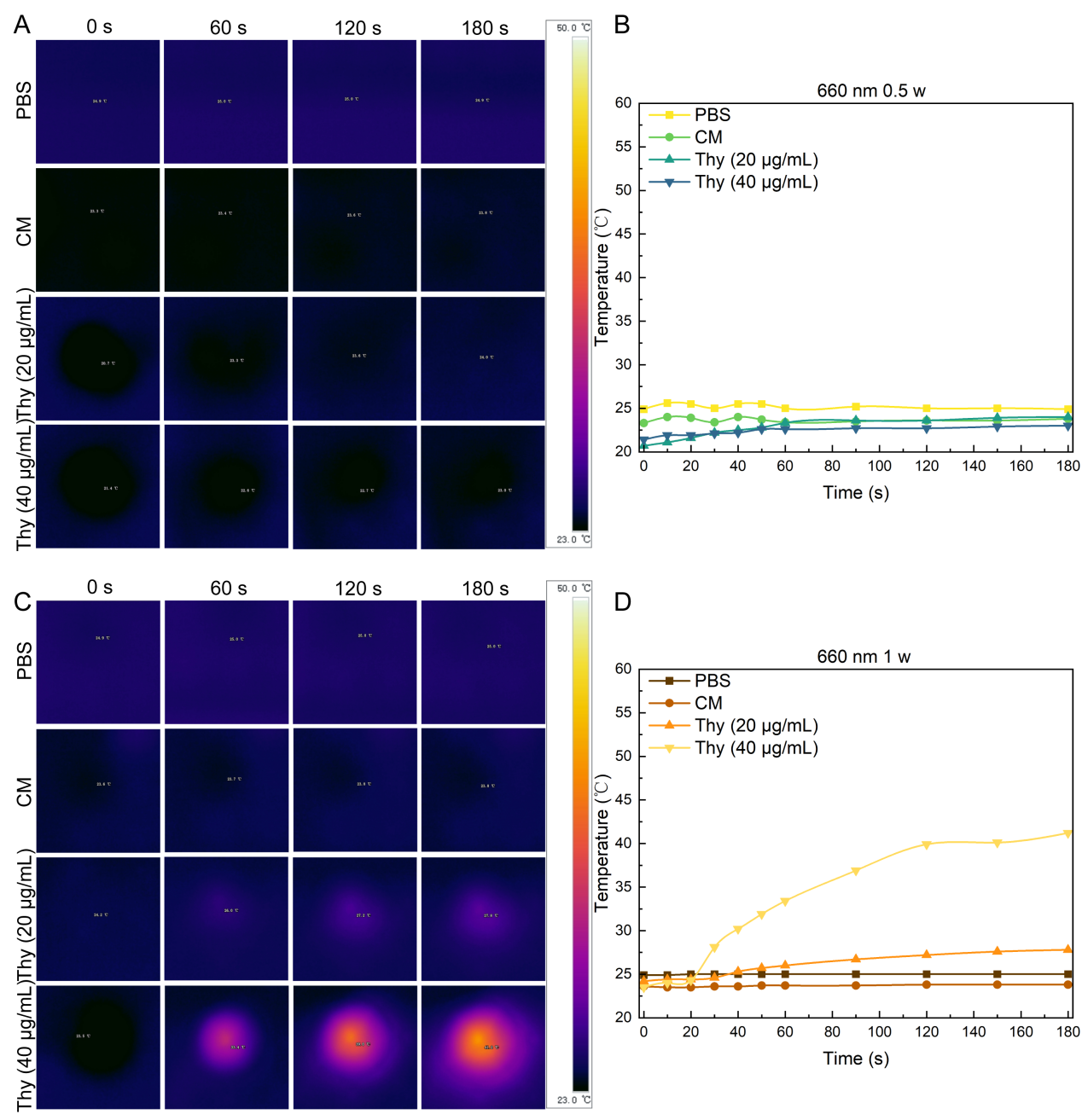


Figure S7 Characterization of photothermal effects of various samples under 660 nm laser irradiation with different power densities. A: Time-dependent thermal images of PBS, CM, Thy 20 μg/mL and Thy 40 μg/mL at 0, 60, 120 and 180 s under 660 nm laser irradiation at 0.5 W. B: Corresponding temperature-time curves of each group in Figure A. C: Time-dependent thermal images of the same groups of samples under 660 nm laser irradiation at 1 W (time points consistent with those in A). D: Corresponding temperature-time curves of each group in Figure C. The color scale on the right side of the thermal images in the figure represents the temperature gradient. The results indicate that the photothermal effect of Thy is dependent on laser power and concentration, while PBS and CM exhibit no obvious photothermal activity.


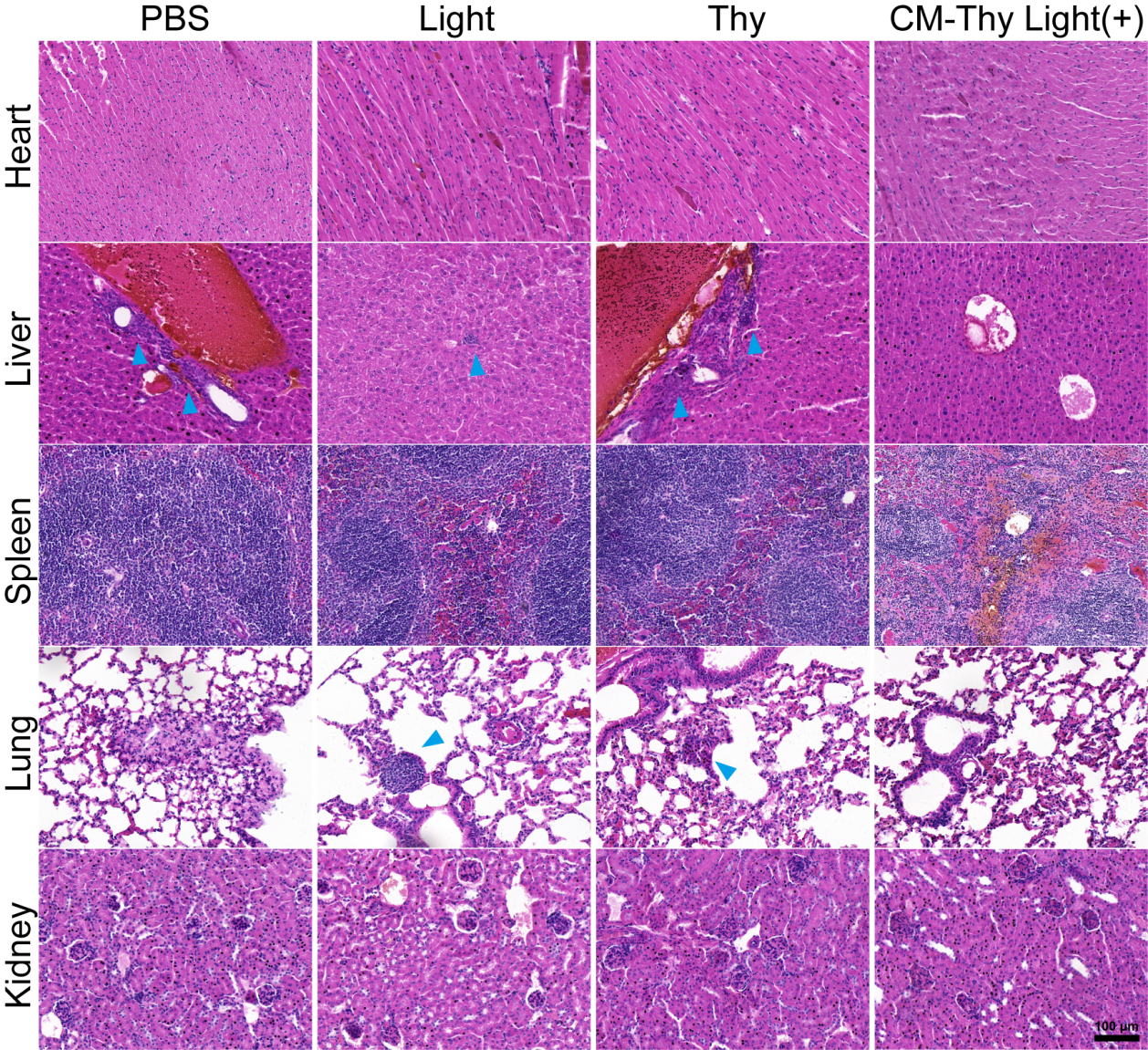


Figure S8 The results of H&E staining of the heart, liver, spleen, lung and kidney in the animals of each experimental group. The blue arrow indicates the tumor tissue.


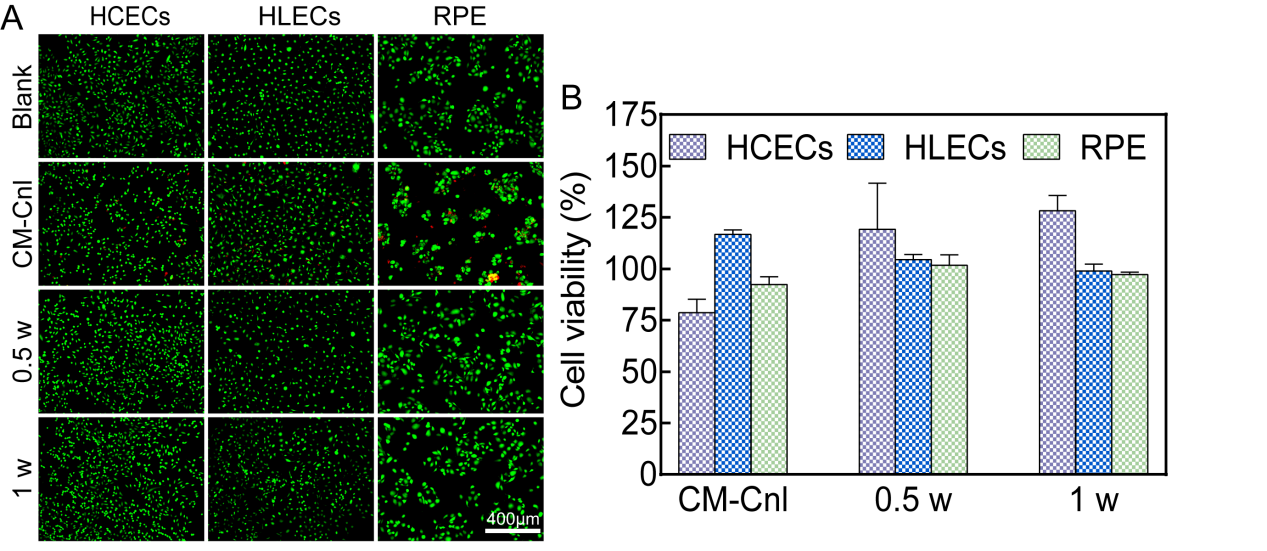


Figure S9 Evaluation of in vitro biocompatibility of CM-Thy and laser irradiation factors in three normal cells in the eye. A: Qualitative analysis of the survival situation of intraocular cells (HCECs, HLECs, and RPE) treated with CM-Thy or laser irradiation (0.5 W/cm^2^ and 1 W/cm^2^). Magnification: 10×. B: Cell viability of three types of intraocular cells treated with CM-Cnl or laser irradiation was quantitatively measured by CCK8 (n=5).


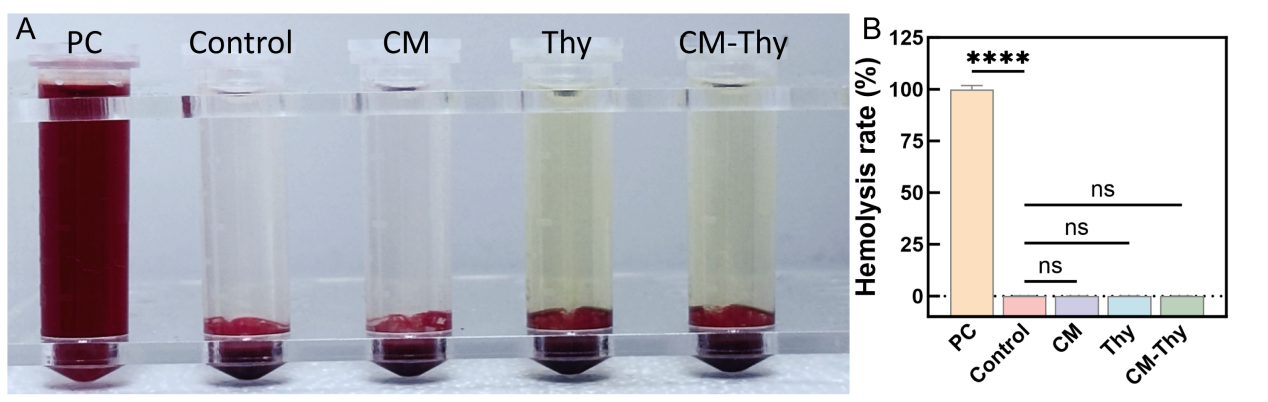


Figure 10 (A) Hemolysis assay and (B) hemolysis rate of CM-Thy (n=5).
